# Robust Photodynamic Therapy Using 5‐ALA‐Incorporated Nanocomplexes Cures Metastatic Melanoma through Priming of CD4^+^CD8^+^ Double Positive T Cells

**DOI:** 10.1002/advs.201802057

**Published:** 2019-01-20

**Authors:** Zhi Li, Cuifeng Wang, Huihui Deng, Jiamin Wu, Huan Huang, Ran Sun, Hongbo Zhang, Xiaoxing Xiong, Min Feng

**Affiliations:** ^1^ School of Pharmaceutical Sciences Guangdong Provincial Key Laboratory of New Drug Design and Evaluation Sun Yat‐sen University Guangzhou 510006 P. R. China; ^2^ Department of Neurosurgery Zhujiang Hospital Southern Medical University Guangzhou 510282 P. R. China; ^3^ Central Laboratory Renmin Hospital of Wuhan University Wuhan 430060 P. R. China

**Keywords:** 5‐aminolevulinic acid, advanced melanoma, CD4^+^CD8^+^ double positive cells, cure, relapse‐free survival

## Abstract

Advanced melanoma can rarely be cured. Photodynamic therapy (PDT) readily eradicates the primary melanoma but has limited ability to destroy the spreading tumor cells unless supported by other combinative interventions to augment systemic antitumor immunity. Based on the previously synthesized penetration‐enhancing biomaterials, a topically administered nanoformulation is developed, which profoundly assists 5‐aminolevulinic acid (5‐ALA) in circumventing skin barrier to be selectively delivered to tumor cells. After endocytosis, accumulated 5‐ALA is efficiently metabolized to a photosensitizer protoporphyrin IX (PpIX) which stimulates a large production of cytotoxic reactive oxygen species (ROS) under illumination. Accompanied by the robust inflammatory responses followed by primary tumor destruction, CD4^+^CD8^+^ double positive T cells are highly boosted to harness host immunity to purge metastases in lymphoid organs. Compared with dacarbazine and programmed death 1 (PD‐1) antibody, this treatment in advanced melanoma murine models, achieves a striking curable rate of 90% without melanoma prognostic markers LDH and S‐100B detection, followed by a relapse‐free survival rate of 83.33% in 300 days. Moreover, the cured mice's immune system function recovers to an extent similar to healthy mice without prolonged or exaggerated inflammation. This study using the synergistic biomaterials approach may thus render 5‐ALA‐mediated PDT a potentially curative therapy for advanced melanoma in clinic.

## 1 Introduction

Malignant melanoma causes most of skin cancer related deaths, although it accounts for only 4% of skin cancers diagnosed.^1^ At very early stage, surgical resection can reach more than 90% curable rate; however, it is very difficult to effectively cure the melanoma once distant metastases occur. Hence, various therapeutic strategies including chemotherapy, radiation therapy, targeted therapy, immunotherapy, and combinations of different types of treatments were employed to treat the advanced‐stage melanoma.^2^ Conventional surgery and chemotherapy normally elicit immunosuppressive effects that allow cancer cells to evade immune surveillance. Besides of the growing success seen with targeted therapies such as serine/threonine‐protein kinase B‐raf (BRAF) and mitogen‐activated protein kinase kinase (MAP2K, also called MEK) inhibitors,^3^ effective immunotherapeutic approach has greatly revolutionized the melanoma treatments including blockade of immune‐inhibitory receptors on activated T cells; for example, using monoclonal antibodies against cytotoxic T‐lymphocyte‐associated antigen 4 (CTLA‐4), PD‐1, and programmed death ligand 1 (PD‐L1).^4^ In terms of survival outcomes, immunotherapy promises to be most significant than any other forms of treatment when tumor metastasis occurs. However, there still exist several obstacles in the field of immunotherapy, such as therapeutic resistance and affordability. In addition to limited success only occurring among few immune responsive melanoma patients, immunotherapy may induce severe autoimmunity against normal tissues.^5^ High cost will weigh heavily on health‐care systems that are already overburdened, in part by the cost of cancer care. Therefore, it still opens the opportunities to develop an alternative patient‐sparing and economic strategy to treat advanced melanoma.

Photodynamic therapy (PDT) uses nontoxic photosensitizers (PSs) that are activated by absorption of light and becomes PSs' triplet, which reacts with molecular oxygen to produce cytotoxic reactive oxygen species (ROS) to kill cancer cells.^6^ More importantly, locally destroyed cancer cells would stimulate the host immune system to suppress metastatic tumor growth, which has been observed in many animal models and patients.^7^ However, PDT‐induced immunostimulatory effects also strongly link to acute inflammation associated with tumor damage. Despite advances in nonmelanoma skin cancer treatment, the use of PDT for melanoma has not yet been substantially pursued, mainly due to its effectiveness limited by poor light penetration depth and lack of ideal PSs. 5‐Aminolevulinic acid (5‐ALA) is a natural safe precursor molecule of the mitochondrial heme synthesis pathway and is enzymatically converted into active photosensitizer protoporphyrin IX (PpIX). However, high polarity of 5‐ALA limits itself to overcome various biological barriers, including stratum corneum and cell membrane. Although employment of lipophilic derivatives of 5‐ALA partly improved skin penetration, these prodrugs are mostly inefficiently cleaved into 5‐ALA.^8^ Thus, challenges in 5‐ALA low bioavailability are primarily required to address for better clinical outcomes against melanoma.

Poly(amidoamine) dendrimers generation two (PAMAM‐G2) are effective skin‐penetration enhancers as well as 5‐ALA delivering vehicles,^9^ while hyaluronic acid (HA) has been commonly considered as a favorable topical vehicle for transdermal drug delivery due to its ability to hydrate stratum corneum.^10^ In addition, α‐cyclodextrins (α‐CDs) have also been demonstrated to stabilize the drug and improve skin permeation.^11^ In our previous works,^12^ PAMAM‐G2 conjugated with α‐CD, as named as CDG2, was able to successfully deliver anionic therapeutic compounds (i.e., DNA) to various cells. Since 5‐ALA exists as a charged zwitterion and HA is rich of negative charges under physiological pH, we propose to utilize cationic CDG2 in combination with HA to encapsulate 5‐ALA with an aid of electrostatic interaction for efficient transdermal delivery and photodynamic therapy in attempt not only to ablate local melanoma, but also to exert immunostimulatory effect to regress the distant metastasis. In the present work, we show that 5‐ALA loaded in CDG2/HA‐constructed nanoparticles (CAH) facilitate their tissue penetration and cellular entry, and selectively induce melanoma cells death owing to excess ROS generation in malignant tissues beyond normal tissues. Compared with free 5‐ALA, PD‐1 antibody and clinical first‐line chemical drug dacarbazine (DTIC), CAH‐mediated PDT significantly increases curable rate and relapse‐free survival rate in advanced melanoma murine models through long‐term regulation of immune system in the presence of CD4^+^CD8^+^ double positive cells. Our results demonstrate that CAH represents a promising easy‐handle photodynamic formulation while providing a safe and cost‐effective optional treatment to fight off metastatic melanoma compared with immunotherapy.

## 2 Results

### 2.1 5‐ALA Incorporated into Nanoscale CDG2/HA Complexes Enhanced Intracellular PpIX Accumulation via Endocytosis

**Figure 1:**
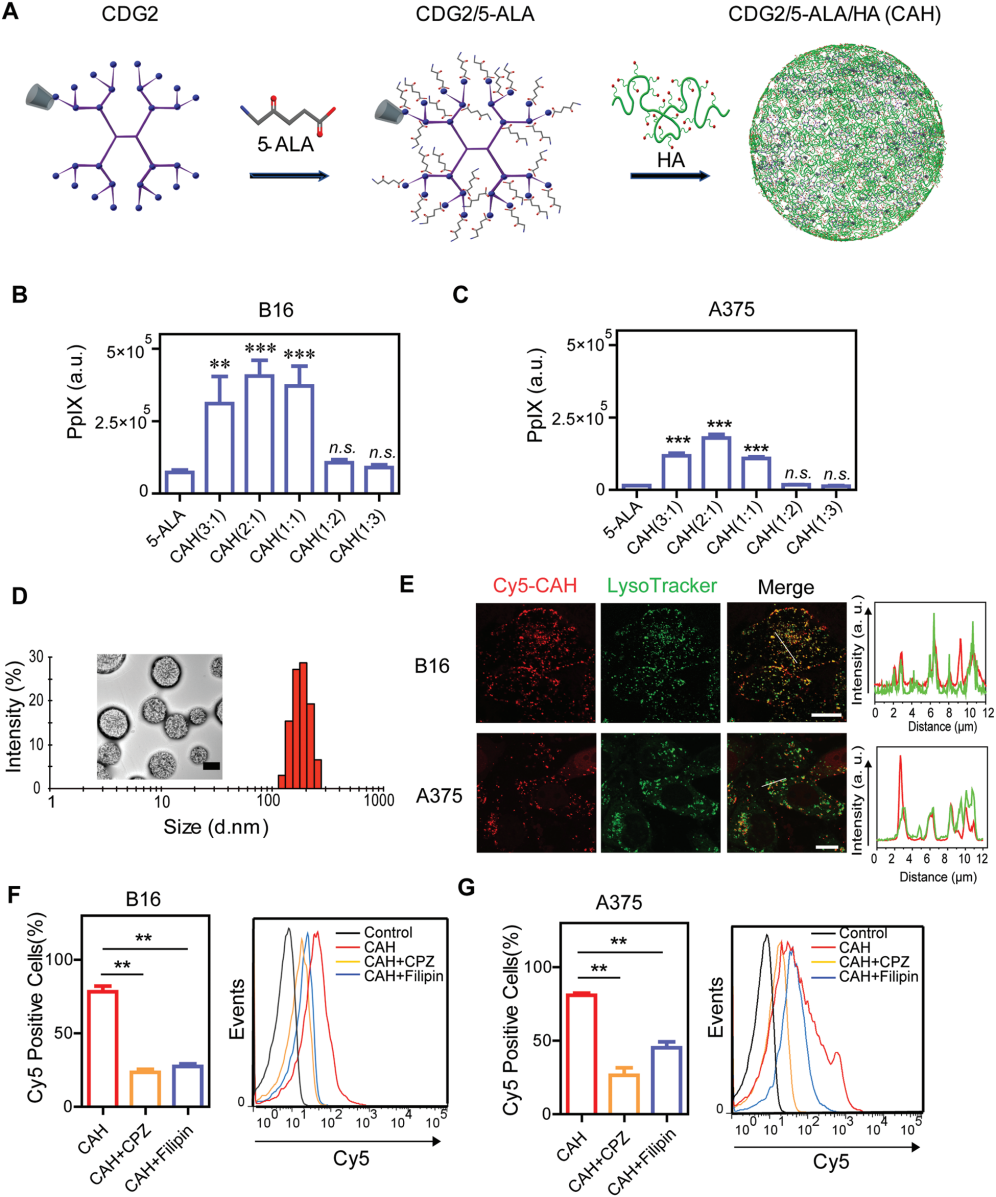
CAH altered cell entry route of 5‐ALA to endocytosis leading to enhanced PpIX accumulation. A) Schematic illustration showing the preparation of CAH complexes. B,C) Fluorescence intensity of PpIX produced by various CAH complexes at varying molar ratios of amine to carboxyl in B16 and A375 melanoma cells, was measured in comparison with that of free 5‐ALA. D) A histogram of CAH (2:1) particle size distribution was generated by dynamic light scattering. Embedded TEM image displayed morphology of CAH (2:1) complexes. Scale bar was 150 nm. E) Intracellular localization of Cy5‐labeled CAH (red). Endo‐lysosomes were stained with LysoTracker (green). Line‐scan graphs in the right panel showed the fluorescence intensity of Cy5‐CAH (red) and endo‐lysosomes (green) along the freely positioned lines (white) in the merged images. Scale bar was 10 µm. F,G) FACS analysis of B16 cells and A375 cells incubation with Cy5‐labeled CAH for 4 h in the absence or presence of endocytic inhibitors CPZ (20 × 10^−6^ m) and filipin (10 µg mL^−1^). The results were reported as the mean ± S.D. of at least three independent experiments, **p < 0.01; ***p < 0.001; n.s. not significant.

CDG2 was synthesized from PAMAM‐G2 and α‐CD via a two‐step reaction mediated by crosslinker *N*,*N*ʹ‐carbonyldiimidazole (CDI) (Figure S1, Supporting Information). The chemical structure of CDG2 was characterized by ^1^H NMR (Figure S2, Supporting Information) and elemental analysis (Table S1, Supporting Information), while its molecular weight was further calculated as 6551 g mol^−1^ on the basis of the established structure of PAMAM‐G2 and α‐CD. It was estimated that the molar ratio of PAMAM‐G2 to α‐CD is 1.7:1. Next, CDG2 was spontaneously assembled with 5‐ALA and HA driving by electrostatic force between protonated amine groups of CDG2 and deprotonated carboxyl groups of HA in combination with 5‐ALA, since 5‐ALA and HA at physiological pH are net negatively charged (**Figure** **1**A). Therefore, various CDG2/5‐ALA/HA complexes, as named as CAH, were formed at the varying molar ratios of peripheral amine to carboxyl from 3:1 to 1:3. In order to optimize formulations, particle characterizations, 5‐ALA encapsulation efficiency and drug loading were measured. While keeping the constant amounts of CDG2 and 5‐ALA in the formulations, along with the decreasing amine/carboxyl molar ratios by adding HA, it showed a smaller particle size and a narrower particle distribution along with a surface charge decrease (**Table** **1**). However, addition of HA induced the 5‐ALA competitively dissociated from polyelectrolyte complexes as evidenced by a dramatic reduction in 5‐ALA encapsulation and loading efficiency. Notably, CAH with an amine/carboxyl molar ratio of 2 (CAH (2:1)) showed the maximal cellular fluorescence intensities in both murine B16 and human A375 melanoma cell lines, reflecting the significantly highest fluorescent PpIX generation in comparison with other compositions (Figure 1B,C). Besides, transmission electronic microscope (TEM) images showed CAH (2:1) nanoparticles are fairly spherical and sub‐200 nm in size, which was in agreement with another measurement provided by dynamic light scatting (Figure 1D). Thus, CAH (2:1) was selected as an optimum formulation for further investigation.

**Table 1:** Characterizations of CAH at the varying molar ratios of peripheral amine to carboxyl groups

	*Z*‐average [*d*, nm]	Zeta potential [mV]	PDIb)	EE%c)	DL%d)
CAH (3:1)a)	275.66 ± 7.78	25.83 ± 1.00	0.29 ± 0.05	75.30%	24.42%
CAH (2:1)	171.00 ± 2.85	23.03 ± 0.40	0.18 ± 0.07	67.28%	19.78%
CAH (1:1)	161.20 ± 5.98	18.27 ± 0.40	0.14 ± 0.06	56.40%	13.60%
CAH (1:2)	164.96 ± 1.23	0.33 ± 0.15	0.16 ± 0.10	50.32%	7.38%
CAH (1:3)	121.80 ± 2.85	−30.33 ± 0.72	0.14 ± 0.12	29.65%	3.29%

^a)^ All measurements were performed in triplicate

^b)^ PDI, polydisperse index

^c)^ EE (%), entrapment efficiency (%)

^d)^ DL(%), drug loading (%).

It has already been reported that cell entry of 5‐ALA may be partially mediated by passive diffusion or some active transporters.^13^ Intriguingly, PpIX production by CAH was significantly greater compared with free 5‐ALA, reflecting enhanced cell penetrating ability of CAH (Figure 1B,C). One possible reason for this enhancement was that CAH might switch to alternative cellular uptake pathways which differed from that of free 5‐ALA. In attempt to understand the underlying mechanism, flow cytometry and confocal laser scanning microscopy (CLSM) analyses were used to detect the internalization efficiency in B16 and A375 cells exposed to cy5‐labeled CAH nanoparticles for 4 h. It was observed that almost all of cy5‐CAH (red) appeared to colocalize with LysoTracker (green). Furthermore, line‐scan analysis also confirmed a large extent of overlap between Cy5‐labeled CAH and LysoTrakcer intensity peaks, suggesting CAH mostly accumulated in endo‐lysosomal compartments (Figure 1E). Chlorpromazine (CPZ) and filipin were extensively used to specifically inhibit clathrin‐ and caveolae‐coated pit endocytosis, respectively. Cellular uptake of CAH in B16 and A375 cells were dramatically reduced by 40–70% in the presence of CPZ and filipin (Figure 1F,G). Collectively, it could be inferred from these data that internalization of nanosized CAH in melanoma cells was mainly mediated by clathrin‐ and caveolae‐dependent endocytosis, which differed from nonendocytotic pathways of free 5‐ALA. Consistently, previous published works also showed that nanoparticles‐based 5‐ALA delivery systems took advantages of endocytic pathways to overcome cellular barriers.^14^ Most importantly, alterations in cell entry pattern contributed to the robust production of PpIX, probably owing to increased 5‐ALA cellular accumulation.

### 2.2 Optimized CAH‐Mediated PDT Specifically Triggered Apoptosis in Melanoma Cells as a Result of Overproduced ROS

**Figure 2:**
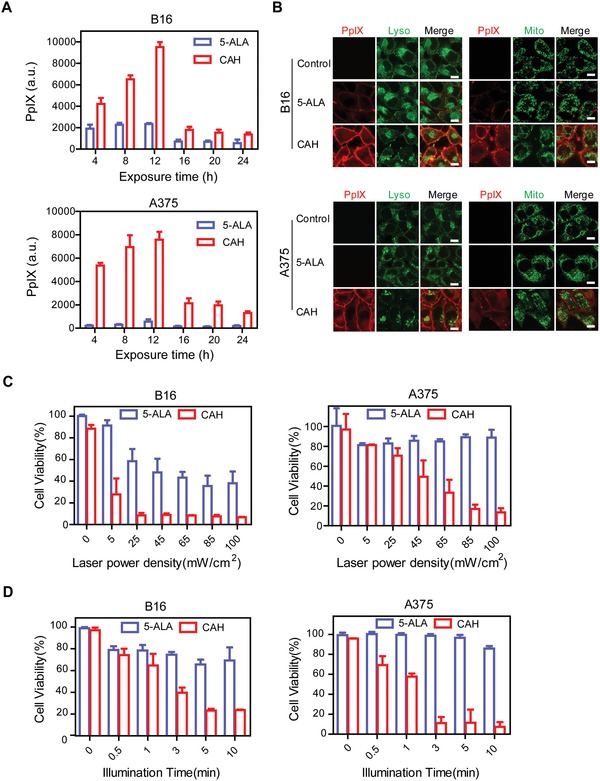
Optimization of CAH‐based photodynamic therapy conditions. A) Fluorescent intensity of PpIX was measured in B16 cells and A375 cells upon exposure to 5‐ALA or CAH containing equal amounts of 5‐ALA (8 µg mL^−1^) for a period of 24 h. B) Intracellular localization of PpIX (red) derived from 5‐ALA and CAH. LysoTracker (Lyso, green) and MitoTracker (Mito, green) were used to stain the endo‐lysosomes and mitochondria, respectively. Scale bar was 10 µm. C,D) Inhibitory effects of light does and illumination time on cell viability of B16 cells and A375 cells treated with 5‐ALA or CAH were evaluated by MTT assay. All data were reported as the mean ± S.D. of three independent experiments.

To further determine the optimal time for irradiation, in vitro release profiles of 5‐ALA from CAH were first monitored in different pH buffers over a period of 24 h. It demonstrated that CAH formulation was able to control 5‐ALA release at a constant rate, which might contribute to maintaining drug concentration in target sites at the efficacious level (Figure S3, Supporting Information). Notably, CAH significantly accelerated drug release at *t* = 12 h in the acidic environments with a pH of 5.5 (mimic endo/lysosomes). Subsequently, a time course of PpIX fluorescent intensities was evaluated in B16 and A375 cells upon exposure to CAH complexes containing 8 µg mL^−1^ of 5‐ALA. An equivalent dose of free 5‐ALA was used as a control. The comparison of the relative fluorescence intensities of PpIX derived from free 5‐ALA or CAH clearly demonstrated the delivery advantages of CAH system. (**Figure** **2**A), which was consistent with previous data (Figure 1B,C). Moreover, PpIX production by CAH displayed a time‐dependent pattern. The levels of fluorescence reached the maximum within 12 h of exposure, and then appeared to decrease afterward. In addition, fluorescence microscopic observation revealed that the accumulated PpIX (red) mainly localized in plasma membrane at *t* = 12 h (Figure 2B). However, there was no fluorescence signal detected in mitochondria which were visualized by MitoTracker staining (green), although PpIX has been reported to generate from 5‐ALA within the mitochondria matrix space.^8^ Likewise, PpIX was barely found in endocytic compartments stained with LysoTracker (green), although CAH entered melanoma cells via endocytosis. Collectively, these data implicated that CAH successfully escaped from endo‐lysosomal entrapments to cytosol within 12 h, and liberated 5‐ALA to access mitochondria for PpIX formation. It was more likely that newly made PpIX was able to redistribute rapidly within the cytosolic membrane due to its high lipophilicity.^15^ Since phototoxicity levels correlated with PpIX abundance, B16 and A375 cells upon exposure to CAH or 5‐ALA for 12 h were chosen for further photodynamic therapy.

**Figure 3:**
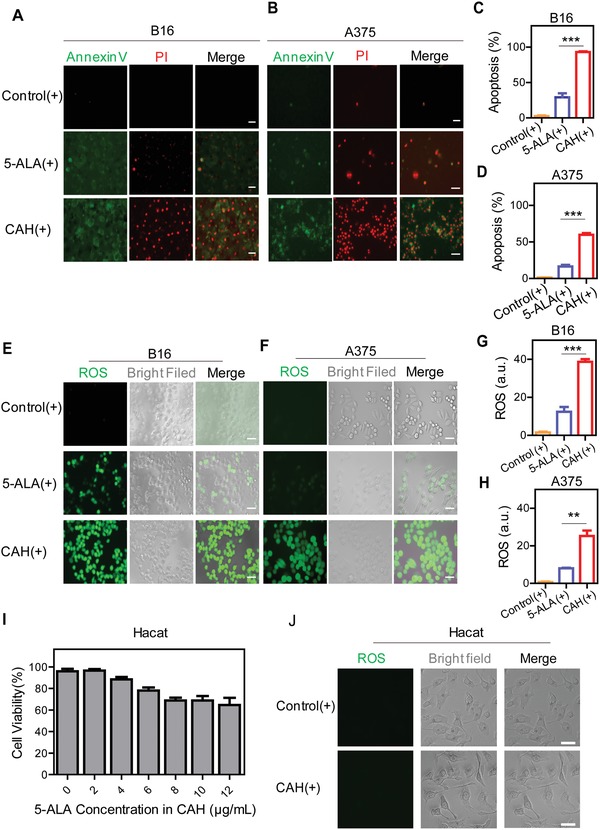
CAH specifically induced melanoma cells apoptosis in correlation with elevated intracellular ROS levels. Melanoma cell lines B16 cells and A375 cells were treated with 5‐ALA (+) or CAH (+) under their optimal PDT conditions. Afterward, the cells were incubated with Annexin V‐FITC (green)/PI (red), followed by A,B) microscopic observation and C,D) FACS analysis. The symbols of addition (“+”) represented the addition of light illumination. In addition, cellular ROS levels (green) of photodamaged B16 cells and A375 cells were monitored using DCFH‐DA assay with E,F) a fluorescent microscope and G,H) a fluorometer. I) After treated with CAH (+) containing various concentrations of 5‐ALA for 12 h, photocytotoxicity of normal skin cell line HaCaT cells at a laser power density of 100 mW cm^−2^ was evaluated by MTT assay. J) ROS production by CAH (+) containing 8 µg mL^−1^ 5‐ALA under 100 mW cm^−2^ was detected. All scale bars were 50 µm. All results were representative of three independent experiments, and shown as the mean values for four samples ± S.D.

Subsequently, methylthiazolyldiphenyl‐tetrazolium bromide (MTT) assay was utilized to evaluate the effects of both energy dose and illumination time on photodynamic efficacy. Given with various light doses, cell viability of B16 cells and A375 cells were measured after 12 h postphototherapy. First of all, we found that no light toxicity was observed in B16 and A375 cells in the absence of CAH or 5‐ALA in the light power range between 5 and 100 mW cm^−2^ (Figure S4A,B, Supporting Information). Additionally, it was also confirmed that either CAH or 5‐ALA without light irradiation barely killed the melanoma cells (Figure S5, Supporting Information). In order to reach more than 80% of cell mortality, therefore, optimum light doses and illumination time were selected as 25 mW cm^−2^ for 5 min for B16 cells and 85 mW cm^−2^ for 3 min for A375 cells, respectively (Figure 2C,D). Apoptotic melanoma cells were further assessed by double‐staining with Annexin V‐FITC/propidium iodide (PI) (Annexin V‐FITC/PI). When B16 or A375 cells were treated with CAH prior to light irradiation, there were only neglectable Annexin V (green) or PI (red) positive cells visualized, suggesting no occurrence of programmed cell death (Figure S6A,B, Supporting Information). Upon exposure to light, however, a large number of green‐red colored cells were observed after the pre‐incubation of CAH, indicating enhanced cells apoptosis (**Figure** **3**A,B). Moreover, fluorescence‐activated cell sorting (FACS) analysis confirmed that CAH‐mediated PDT induced 93.08 ± 1.28% of B16 cells and 59.83 ± 4.08% of A375 cells undergoing apoptotic death (Figure 3C,D and Figure S6B, Supporting Information). This represented a relative 3.2–3.6‐fold increase compared with free 5‐ALA under the same illumination conditions. Therefore, these data implied that CAH‐induced apoptosis was triggered by the availability of light source. In addition, PpIX‐induced oxidative damage has been considered as a main cause for cell apoptosis and necrosis.^16^ Indeed, intracellular ROS production (green) in CAH‐incubated melanoma cells were observed to be significantly augmented in a light‐dependent manner compared with the cells treated with 5‐ALA (Figure 3E–H and Figure S7, Supporting Information). By contrast, normal skin cells HaCaT showed remarkably high tolerance to CAH‐induced phototoxicity even at the maximum light power density of 100 mW cm^−2^ (Figure 3I). In consistence with previous studies that intracellular accumulation of 5‐ALA‐derived PpIX was more pronounced in neoplastic cells as compared to normal cells,^8^ there was negligible ROS produced in normal cells HaCaT (Figure 3J); Therefore, it could be inferred that light‐activated CAH was able to selectively destroy the target tumor, while sparing normal skin tissues from apoptosis induction.

### 2.3 Establishment of an Advanced Melanoma Murine Model

**Figure 4:**
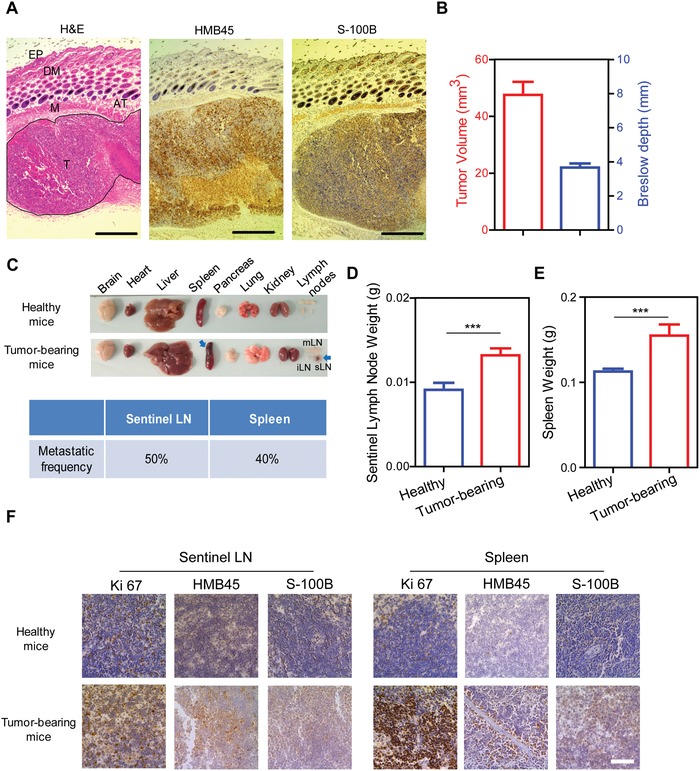
Establishment of advanced melanoma murine models. A) Longitudinal sections of subcutaneous melanoma and adjacent skin were analyzed by hematoxylin and eosin (H&E) staining, and immunohistochemical staining for HMB45 and S‐100B. EP, Epidermis; DM, Dermis; M, Muscle; AT, Adipose tissue; T, Tumor. Scale bar was 1 mm. B) Tumor volume and Breslow depth of subcutaneous melanoma were measured. C) Photographs of dissected organs from healthy mice and tumor‐bearing mice (upper panel). mLN, mesenteric lymph nodes; iLN, tumor‐distant inguinal lymph nodes; sLN, tumor‐sentinel inguinal lymph nodes. Arrows indicated the melanoma metastasis nodules in sentinel lymph nodes and spleen, and their metastatic frequency were further calculated. D–F) Sentinel lymph nodes and spleens were weighted and stained with Ki67, HMB45 and S‐100B. Scale bar was 50 µm. *n* = 10 mice per group. All data were expressed as mean ± S.D., ***p < 0.001.

To test in vivo anti‐tumor efficacy of CAH, B16 subcutaneous melanoma model was first established. After being allowed to form a palpable tumor besides hind limbs with an average volume of 47.46 ± 14.56 mm^3^ (*n* = 10) in 3 days, the mice were sacrificed and tumors with adjacent tissues were excised to predict melanoma progression. Hematoxylin and eosin (H&E) stained longitudinal sections showed that tumors aggressively invaded throughout dermis and spread into the muscle layer. Furthermore, classical melanoma markers human melanoma black (HMB)‐45 and S100 calcium‐binding protein B (S‐100B) diagnosed the same lesions as melanocytic neoplasms (**Figure** **4**A). According to Breslow scales, the distances between the upper layer of the epidermis and the deepest point of tumor penetration was measured as 3.26 ± 1.03 mm, which was identified as melanoma stage III (2–4 mm) (Figure 4B). Besides primary tumors, we also collected all other organs and tissues including locoregional lymph nodes to detect whether melanoma cells spread into other parts of body. In contrast to healthy mice, it appeared that pigmented lesions mainly presented in the sentinel lymph node and distant spleen with incidence of 50% and 40%, respectively, in ten mice (Figure 4C). Accompanying with swelling tumor‐draining lymph nodes and spleens, their weights were dramatically increased compared with those of healthy mice, probably due to immune stimulation by antigenicity of invasive tumor cells^17^ (Figure 4D,E). Moreover, immunohistochemical (IHC) assessments of prognostic factor Ki67, HMB‐45, and S‐100B expression further confirmed that melanoma cells invaded in sentinel lymph node and distant spleen (Figure 4F). All these data suggested that the established B16 melanoma models had advanced to a stage III or beyond.

### 2.4 CAH‐Induced Photocytotoxicity Completely Eradicated Primary and Metastatic Melanoma

**Figure 5:**
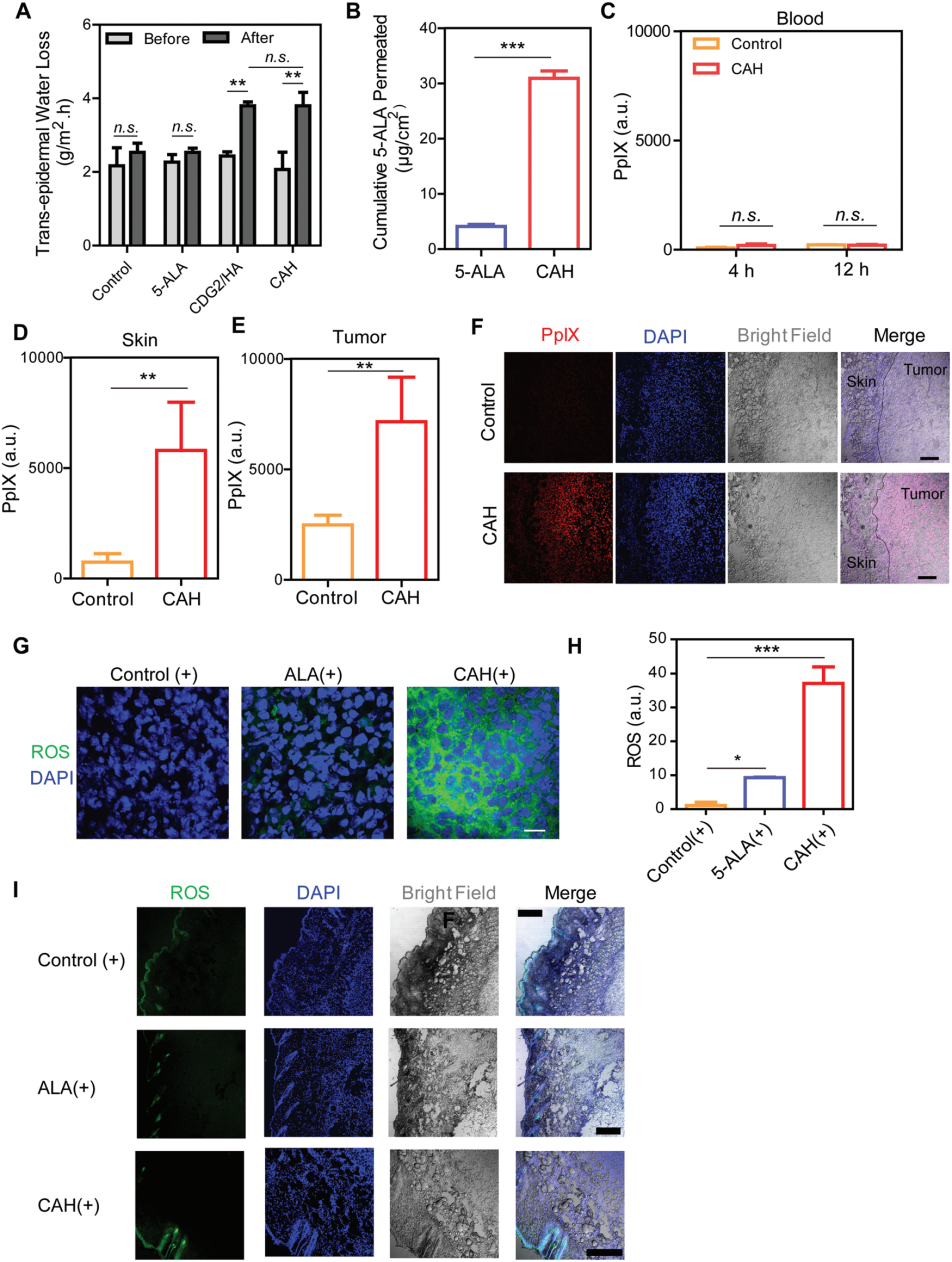
Enhanced skin permeabilization augmented CAH‐derived PpIX production and ROS accumulation in tumor. A) Trans‐epidermal water loss (TEWL) was measured from the mice skin before and 12 h after topically applied with blank cream, 20% w/w 5‐ALA cream, 20% CDG2/HA cream and 20% CAH cream with 5 min of light irradiation. The mice skin applied with blank cream was used as a control. B) Cumulative 5‐ALA amounts penetrated through the ex vivo rat skins were measured within 12 h. C) Blood PpIX‐emitted fluorescence intensity was used to monitor its circulating concentrations after tumor‐bearing mice exposure to 20% CAH cream at 4 and 12 h, respectively. D,E) Skin deposition and tumor accumulation of PpIX were detected with a fluorometer at 12 h after in vivo topical application of 20% CAH cream. F) Distribution of CAH‐derived PpIX in skin and tumor was monitored by CLSM. Scale bar was 100 µm. G,H) After topically applied with blank cream, 20% 5‐ALA cream and 20% CAH cream for 12 h, ROS generation in tumors were triggered under 635 nm laser irradiation (25 mW cm^−2^) for 5 min and further quantified. Scale bar was 20 µm. I) ROS levels in the mice skin sections were detected. Scale bar was 100 µm. All data were shown as mean ± S.D., *n*  =  3, n.s., not significant, *p < 0.05, **p < 0.01, ***p < 0.001.

5‐ALA transdermal delivery by CAH primarily depended on its ability to overcome the skin barrier. Therefore, several ex vivo assays with mice/rats skin were utilized to predict the skin permeability of CAH. Transepidermal water loss (TEWL) was positively correlated with the disruption degree of skin barrier. As depicted in **Figure** **5**A, topical 20% w/w CAH cream or 20% CDG2/HA cream resulted in a significant increase in TEWL as compared to those of the mice treated with blank cream or 20% 5‐ALA, suggesting CDG2/HA formulation readily impaired the skin integrity (Figure 5A). Accompanying with enhancement in skin permeability, therefore, CAH was able to constantly reach deeper tissue layers over time, with a 7.52‐fold increase of 5‐ALA accumulation across the skin with an aid of nanocarriers than free 5‐ALA at *t* = 12 h (Figure 5B). Although ex vivo studies confirmed that CAH improved 5‐ALA to penetrate the skin, we found that circulating PpIX levels were still negligible in vivo (Figure 5C). It is possible that CAH were retained in skin and tumor where highly accumulated PpIX were detected (Figure 5D–F). Consequently, ROS levels were also remarkably elevated in tumor sections after light illumination (Figure 5G,H). On the contrary, negligible ROS production by control (+), 5‐ALA (+), and CAH (+) were only traced in hair follicle niche but not distributed in the whole skin (Figure 5I), suggesting ROS were selectively produced in tumor cells in consistence with in vitro observations (Figure 3). Thus, these data demonstrated that enhanced skin penetration by CAH might facilitate tumor cells uptake photosensitizers leading to high ROS generation, since primary tumor localized from superficial to deeper skin layers (Figure 4A). As melanoma cells exhibited high ROS accumulation which distinguished them from normal skin cells, we concluded that CAH was able to selectively induce apoptosis in melanoma cells in vivo.

**Figure 6:**
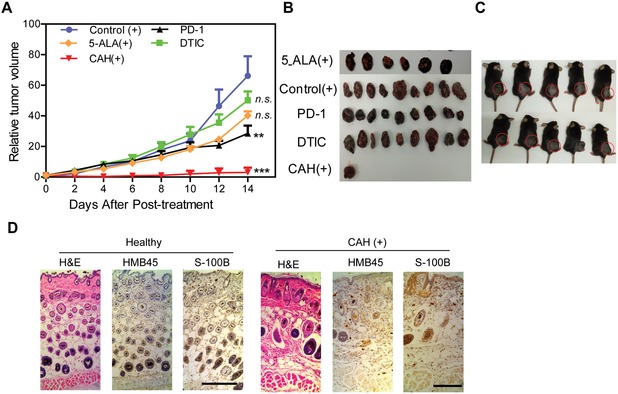
CAH‐assisted PDT therapy completely eradicated primary melanoma and recovered skin barrier function. A) Tumor growth rates were monitored by relative tumor volumes within 14 days after various treatments. Relative tumor volume (RTV) was calculated according to the following formula: RTV = TV*_n_*/TV_0_, where TV*_n_* is the tumor volume at day *n* and TV_0_ is the tumor volume at day 0. Data from experimental were expressed as mean ± S.D., *n*  =  10, *p <  0.05, **p < 0.01, ***p < 0.001 versus control (+). B) Photographic images of B16 tumors dissected from mice followed by experimental termination. C) Photographic images of alive CAH (+)‐cured mice. Red circles indicated tumor ablation zones. D) H&E staining and IHC staining with antibodies to HMB45 and S‐100B reflected CAH (+)‐mediated PDT recovered skin barrier function after removal of tumor lesions. Scale bar was 200 µm.

Next, we tested anti‐tumor effect of CAH on established B16 advanced melanoma mice. Without laser illumination, there was no significant inhibitory effect observed on all the treated mice, whose mortality rates reached 40% (Figure S8, Supporting Information). On the contrary, light exposure exerted a dramatic growth inhibition on CAH‐pretreated mice (**Figure** **6**A,B). Strikingly, CAH with light irradiation (CAH (+)) completely eradicated the primary tumor with a curable rate of 90% (Figure 6C). However, B16 melanoma progressed aggressively in the mice without any treatment, or treated with 5‐ALA (+) or DTIC. Although PD‐1 antibody after i.p. injection for 14 days significantly suppressed tumor growth compared with untreated tumor‐bearing mice, its mortality rate still remained 10%. Compared to healthy mice, H&E staining demonstrated no malignant lesions detected in the mice after CAH (+) treatment, and the skin recovered to normal structure. Consistently, immunostaining for HMB45 and S‐100B further confirmed that primary melanoma was completely ablated by CAH‐mediated PDT therapy (Figure 6D), whereas the remaining uncured mice exhibited varying degrees of tumor progression (Figure S9, Supporting Information).

**Figure 7:**
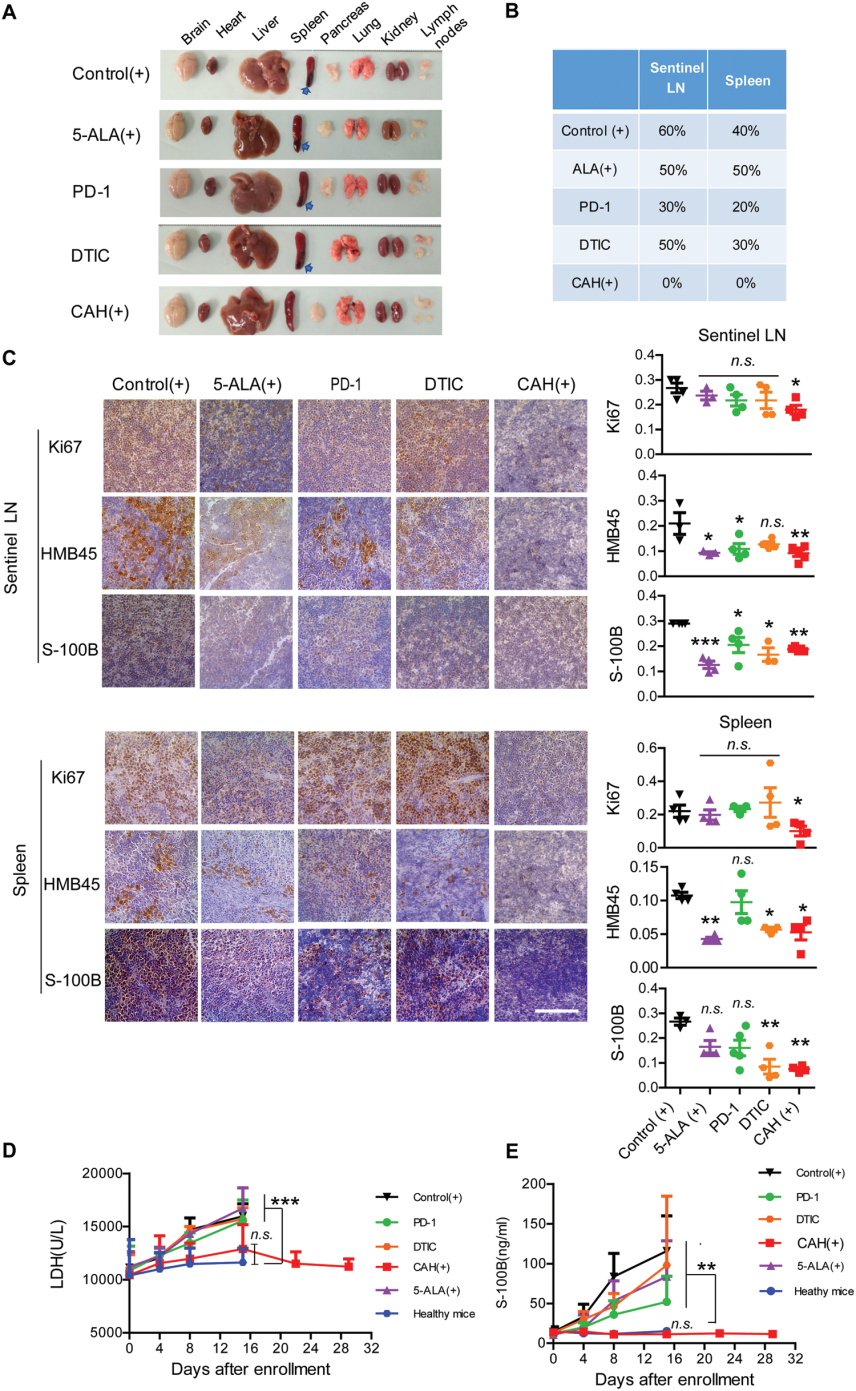
CAH‐mediated PDT remarkably purged metastases in lymphoid organs. A,B) Representative photographs of dissected organs from all the mice over a 14 day treatment period. Arrows indicated the pigmented metastasis nodules, and their metastatic frequency were further calculated and present in the table. *n* = 10 mice per group. C) Immunohistochemical staining for Ki67, HMB45 and S‐100B in sentinel LNs and spleens. Optical density scores were measured by digital image analyses using Fiji program. Four microscopic images were taken randomly from each sample, *n*  =  4 mice per experimental group. Scale bar was 100 µm. Values were expressed as mean ± S.D., n.s., not significant, *p < 0.05, **p < 0.01, ***p < 0.001 versus control (+). D,E) Serum LDH and S‐100B concentrations were measured over time. Data were presented as mean ± S.D., *n* = 10, n.s., not significant, **p < 0.01, ***p <  0.001 versus Healthy mice.

Since B16 melanoma cells frequently spread to regional lymph nodes and distant spleen in our mice model, we further investigated whether CAH effectively restricted the tumor metastases. As a result, no pigmented lesions were observed in sentinel lymph nodes and distant spleens, indicating CAH (+) markedly inhibited tumor cells dissemination in comparison with other treatments (**Figure** **7**A,B). Furthermore, there were no infiltrating tumors found in the lymph nodes and spleen as evidenced by low expression of melanoma markers HMB45 and S‐100B in tissue sections (Figure 7C, left panel), while staining intensity scoring showed strong agreements with our observations (Figure 7C, right panel). Besides, serum S‐100B and lactate dehydrogenase (LDH) concentrations are widely used as prognostic biomarkers in advanced melanoma.^18^ During dosing with various drugs, serum S‐100B and LDH levels in CAH (+)‐treated mice were insignificantly different from those of healthy mice, but were statistically lower as compared to other treatments including PD‐1, DTIC, 5‐ALA (+), and control (+) mice (Figure 7D,E). Intriguingly, withdrawal of CAH (+) for two weeks resulted in S‐100B and LDH remaining at the normal levels. Collectively, these data demonstrated that CAH‐mediated PDT remarkably destroyed the primary tumor, purged the metastatic tumor, and eventually cured the advance melanoma.

### 2.5 CAH (+) Augmented the Frequency of CD4^+^CD8^+^ Double Positive T Cells to Boost Anti‐Tumor Immunity

**Figure 8:**
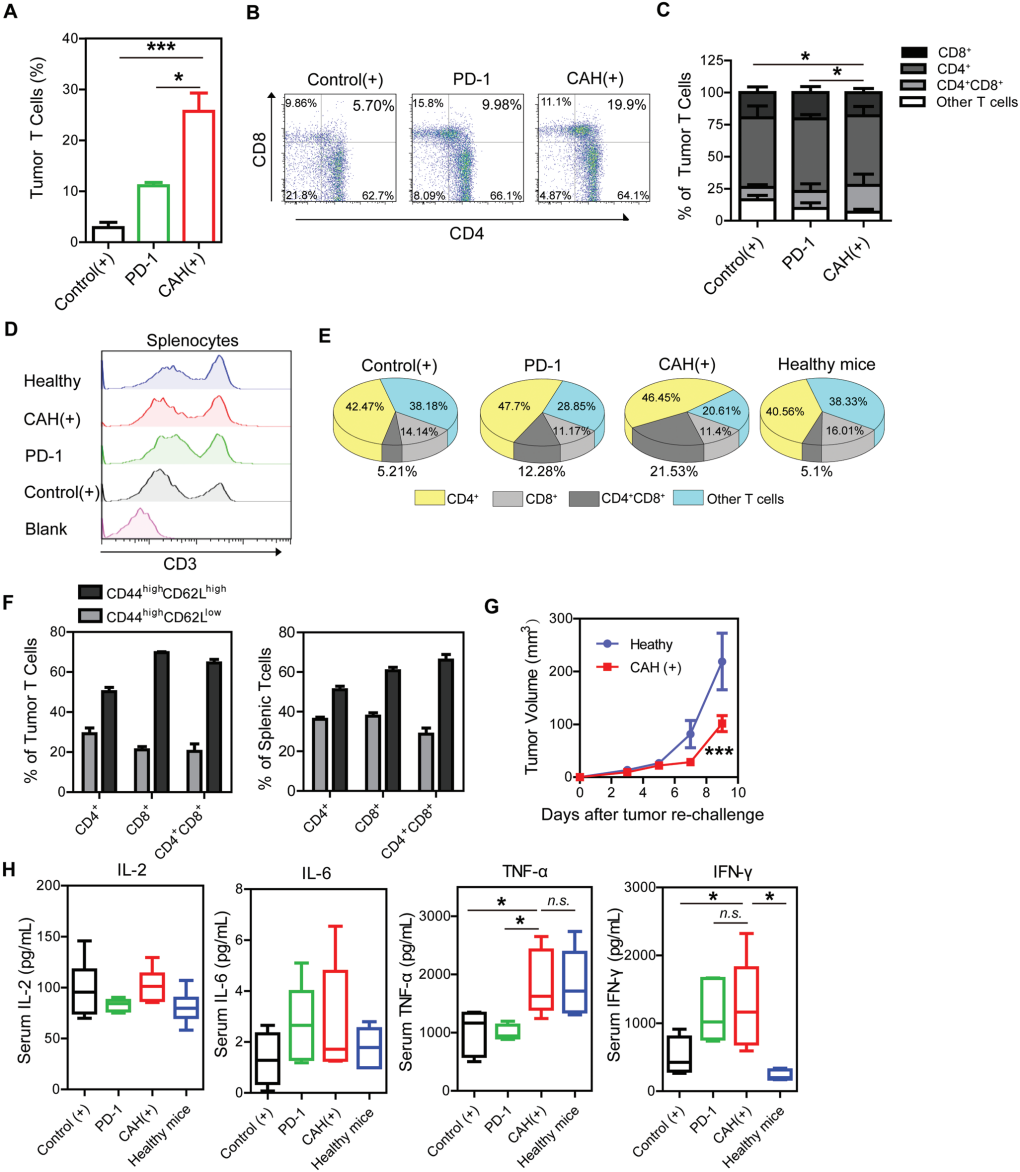
CAH‐mediated PDT highly activated CD4^+^CD8^+^ double positive T cells with elevated proinflammatory cytokines. After two‐circle dosing, fresh excised tumor and spleen from each tested mouse were processed into a single cell suspension, stained with antibodies and analyzed via flow cytometry. A) The percentage of live tumor‐infiltrating T cells marked with CD45^+^CD3^+^ within tumor tissue. B,C) Proportions of CD4^+^, CD8^+^, CD4^+^CD8^+^, and other T cells out of total tumor‐infiltrating T cells. D,E) Splenic T cells were marked with CD3, and their subsets were further determined with CD4 and CD8. F) Phenotypes of CD4^+^, CD8^+^ single T cells, and CD4^+^CD8^+^ double positive T cells with indicated surface marker CD44 and CD62L. G) Five CAH (+)‐cured mice were re‐challenged with 10^6^ of B16 cells on the opposite flank, and five healthy mice of the same age were used as control. Tumor volume was monitored over time. H) Serum cytokines IL‐2, IL‐6, TNF‐α, and IFN‐γ were measured after two‐circle dosing. Data shown as mean ± S.D. were pooled from at least three independent experiments with at least three mice per group. *p < 0.05, ***p < 0.001, n.s. not significant.

Since CAH‐derived PpIX was confined to accumulate in the irradiated area (Figure 5), there must exist other underlying mechanisms of the curable effect of CAH (+) on metastatic melanoma. Increasing evidences showed that PDT‐induced tumor destruction was able to stimulate immune responses which synergized anti‐tumor effect of PDT. Notably, the presence of tumor‐infiltrating T cells strongly correlated with survival outcomes in patients with cancer.^19^ To address whether PDT induced alterations of tumor‐infiltrating T lymphocyte populations, therefore, we next analyzed single cells isolated from tumor tissue after two‐cycle treatments using flow cytometry with previously reported gating strategy (Figure S10, Supporting Information). PD‐1 antibody activated cytotoxic T cells to kill tumor cells and was used as a positive control. CAH (+)‐mediated treatments exhibited ≈8.9‐fold and 2.3‐fold improvements in intratumoral incidence of T cells over control (+) and PD‐1, respectively (**Figure** **8**A). Next, subpopulations of T cells were further assessed with immune markers CD4 and CD8. There was no significant difference in either CD4^+^ or CD8^+^ single positive T cells between different groups (Control (+), PD‐1, and CAH (+)). Intriguingly, tumor CD4^+^CD8^+^ double positive T cells were remarkably accumulated in response to CAH (+), accounting for 21.00 ± 7.08% of all T cells (Figure 8B,C). Consistently with previous studies,^20^ only ≈1–2% of circulating T cells co‐expressing CD4 and CD8 could be detected in peripheral blood of CAH (+)‐treated mice, and displayed the similar phenotyping as other experimental groups (Figure S11, Supporting Information). Although most mature T cells exclusively expressed CD4 or CD8, but not both once leaving thymus, however, the frequency of CD4^+^CD8^+^ double positive T cells could be augmented for adaptive immune response under inflammatory conditions, such as autoimmune diseases, acute viral infections, and cancer.^21^ It might be assumed that influx of CD4^+^CD8^+^ double positive T subsets into a tumor resulted in the induction of an inflammatory microenvironment, which activated anti‐tumor immunity after PDT. In addition, CAH (+) treatment also cleared the existing splenic metastases and prevented melanoma from spreading to other distant organs. Therefore, we further examined T cell populations marked with CD3 in spleens after various treatments. In contrast to an increase of tumor‐infiltrating T cells induced by CAH (+), there was no statistical change in CD3^+^ percentage of splenocytes among different experimental groups (Figure 8D). Compared with CD4 and CD8 counterparts, however, a greater proportion of CD4^+^CD8^+^ T lymphocytes was also highly activated upon exposure to immune stimulation with CAH (+) (Figure 8E). Moreover, memory cell differentiation was performed using CD62L/CD44 phenotypic markers. It showed that activated CD4^+^CD8^+^ double positive T cells in tumor and spleen induced by CAH (+) were mostly memory T cells with mostly being central memory cells (CD44^high^ CD62L^high^ phenotypes) and less being effector memory cells (CD44^high^ CD62L^low^ phenotypes). Similar T cell differentiation was observed within CD4^+^ and CD8^+^ single positive cells (Figure 8F). CAH (+)‐cured mice were resistant to tumor re‐challenge with B16 cells as compared to naïve mice (Figure 8G), suggesting cured mice might develop the long‐lasting immune memory. Thus, it was inferred from above data that CAH (+) treatment might promote the proliferation of CD4^+^CD8^+^ double positive T subsets and boost a systemic and durable immune defense against metastatic melanoma. Notably, CD4^+^CD8^+^ double positive T lymphocytes had been shown to produce pro‐inflammatory cytokines and exert stronger cytotoxic activity compared with CD4^+^ or CD8^+^ single positive T cells.^22^ Subsequently, serum proinflammatory cytokines levels including interleukin 2 (IL‐2), interleukin 6 (IL‐6), tumor necrosis factor α (TNF‐α) and interferon gamma (IFN‐γ) were measured after two‐cycle treatments. Previous studies implicated that IL‐6 expression correlated with T cell proliferation and function,^23^ while IL‐2 played a critical role in the activation of immune system by regulating lymphocytes.^24^ As depicted in Figure 8H, there were no significant alterations between each group with regard to serum IL‐2 and IL‐6 levels, suggesting that IL‐2 and IL‐6 signal transductions were not associated with melanoma progression in this study. However, serum concentrations of TNF‐α and IFN‐γ were elevated in mice receiving CAH (+) in comparison with untreated tumor‐bearing mice. Previously, increased TNF‐α and IFN‐γ levels by PDT had been confirmed to create an immunogenic microenvironment against metastatic cancer.^25^ In addition, CD4^+^CD8^+^ double positive T lymphocytes were correlated with enhanced TNF‐α and IFN‐γ production.^26^ In contrary to CAH (+), PD‐1 induced an enhancement in IFN‐γ secretion but not TNF‐α. Overall, these data demonstrated that CAH‐mediated PDT potentiated local and systemic anti‐tumor immunity by activation of CD4^+^CD8^+^ double T cells accompanied with increased proinflammatory cytokines production.

### 2.6 CAH (+) Recovered Immune System and Prolonged Relapse‐Free Survival

**Figure 9:**
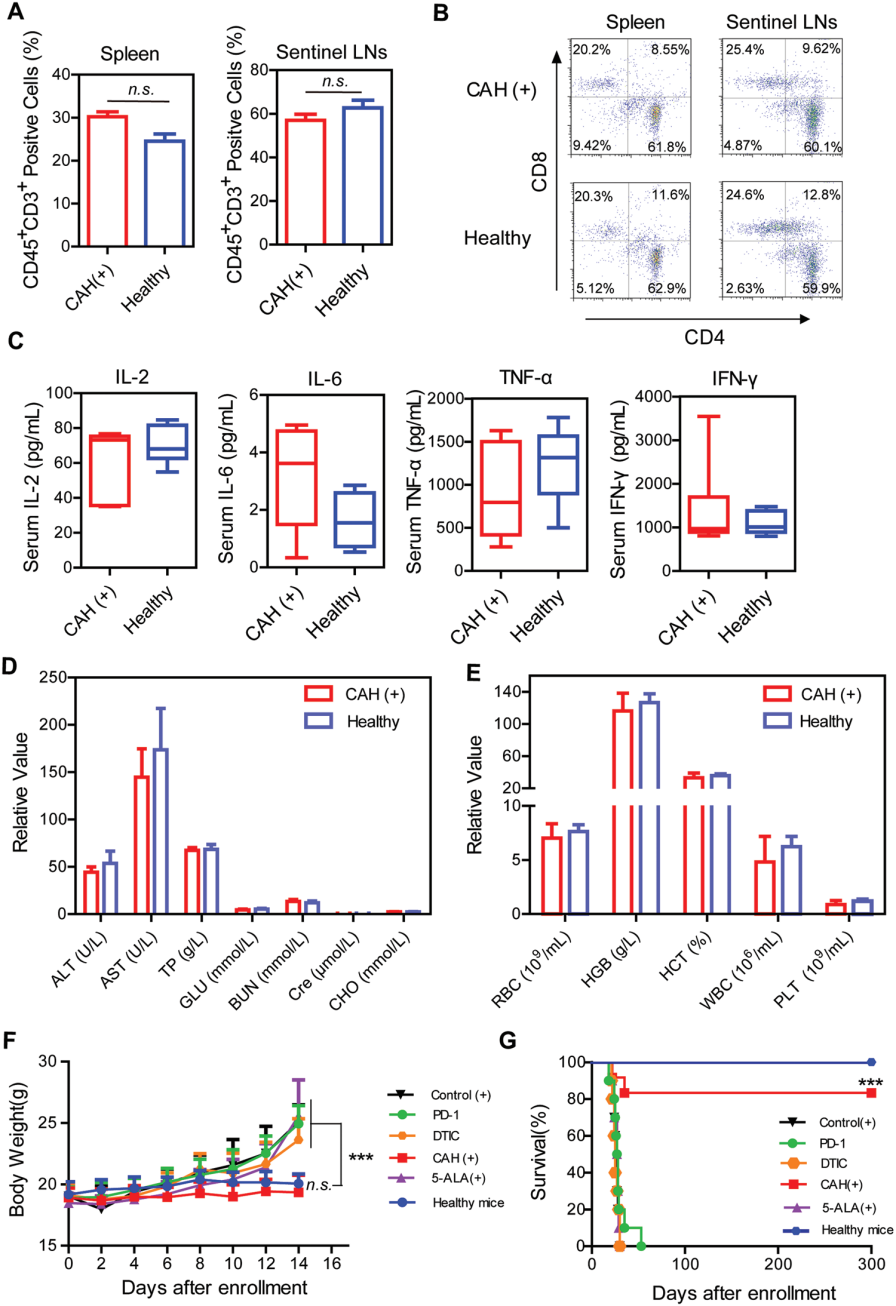
CAH (+) restored immune balance and prolonged relapse‐free survival. A) Percentage of CD45^+^CD3^+^ T cells within spleen and sentinel LNs from CAH (+)‐cured mice and healthy mice. B) T cell subsets were further analyzed with CD4 and CD8 marker. C) Cytokines profiles of CAH (+)‐cured mice and healthy mice. D) Serum biochemistry profile of CAH (+)‐cured mice and healthy mice. ALT, alanine aminotransferase; AST, aspartate aminotransferase; TP, total protein; GLU, glucose; BUN, blood urea nitrogen; Cre, creatinine; CHO, cholesterol. E) Complete blood panel test of CAH (+)‐cured mice and healthy mice. RBC, red blood cell; HGB, hemoglobin; HCT, hematocrit; WBC, white blood cell; PLT, platelet. F) Body weights of mice after various treatments over time were monitored. G) Survival rate experimental mice. Data were representative of three independent experiments with *n* = 3–10 mice per group, as shown as mean ± S.D., ***p < 0.001, n.s. not significant.

Although an effective immune response to eliminate cancer cells was essential, a prolonged or exaggerated response could cause the imbalance in the host immune system. Therefore, we further examined the T cell subpopulations in CAH (+)‐cured mice after seven dosing. In spleen and sentinel lymph node, analysis of T cells (CD45^+^CD3^+^ positive cells) showed no changes compared with healthy mice of the same age (**Figure** **9**A). Additionally, CD4^+^, CD8^+^, and CD4^+^CD8^+^ double positive T cells in lymphoid organs (spleen and sentinel LN) were not persistently activated after treatment completion, since the phenotypic frequencies were comparable with those of healthy mice (Figure 9B). To provide further evidence of host immunity modulated by CAH (+), serum cytokines including IL‐2, IL‐6, TNF‐α, and IFN‐γ were also evaluated, and there was no significant difference observed between CAH (+)‐cured mice and healthy mice (Figure 9C). Additionally, serum biochemistry assay and complete blood panel test were further performed to evaluate the safety profiles of CAH‐mediated PDT. The results demonstrated that CAH did not induce systemic toxicity in terms of blood biochemistry and hematology (Figure 9D,E). Next, body weights were monitored to assess the safety of various treatments. Throughout the test period, there were no significant alterations in body weights of the tumor‐bearing mice after the addition of CAH (+) compared with those of healthy mice. However, body weights of other mice continually increased with time probably due to growing tumor burden (Figure 9F). Most importantly, survival rate of the mice receiving CAH (+) reached 83.33% without recurrence more than 300 days, which was significantly improved than that of the mice treated with PD‐1, DTIC and 5‐ALA (Figure 9G). Collectively, these data indicated that CAH‐mediated PDT harnessed the immune system not only to stimulate anti‐tumor immune response during treatments, but also to restore the immune system function as healthy mice after treatments. Consequently, CAH (+) prolonged the survival rate without tumor relapse.

## 3 Discussion

PDT has great potential to become an ideal oncological intervention for metastatic melanoma which is able to selectively destroy the primary tumor and its microenvironment and at the same time provoke the immune system to attack any remaining or recurring melanoma cells. However, the fact that PDT has yet been accepted as a first‐line clinical option for cancer patients is mainly because of its inefficiency. For example, topically applied 5‐ALA is limited by poor skin penetration and low bioavailability, and only indicated for some nonmalignant skin cancers. Here, we show that 5‐ALA is incorporated into a nanocarrier constituted by CDG2 and HA, as named as CAH. Along with enhanced skin penetration, nanosized CAH facilitates the cellular uptake of 5‐ALA across cell membrane barrier, followed by increased PpIX production. These improvements are partly attributed by skin‐penetrating enhancers in CAH, such as PAMAM‐G2, HA and α‐CD which are previously well‐documented in dermal and transdermal drug delivery.^9^, ^10^, ^11^ Additionally, in contrast to diffusion‐ or transporter‐mediated 5‐ALA cell entry routes, it is more likely that CAH is internalized by melanoma cells via multiple endocytic pathways to increase the cellular accumulation of 5‐ALA, leading to enhanced PpIX formation. Likewise, alterations in internalization pattern also induce intracellular re‐localization of PpIX. In contradiction to some previous reports,^27^ we find CAH‐induced PpIX is largely localized in the plasma membrane instead of mitochondria where 5‐ALA is initially converted into PpIX. It may be inferred that PpIX is formed in mitochondria after 5‐ALA dissociated from CAH, but rapidly diffuses to plasma membrane due to its lipophilicity. This phenomenon may be explained by varying subcellular redistribution of PpIX depending on different incubation time.^28^

Notably, CAH is able to specifically target melanoma cells but spare normal skin cells. CAH shows neglectable dark toxicity and no allergic reactions during its administration. In addition, although CAH‐induced PpIX mainly accumulates in skin and tumors, it rarely accesses circulation in vivo. Only a high yield of cytotoxic ROS is observed in tumor region during illumination, which may be attributed to the fact that cancer cells produce more PpIX in comparison with normal cells.^29^ Moreover, 5‐ALA and other CAH components are easily obtained or produced, which will benefit further scale‐up manufacturing at a lower cost. These features characterize CAH as a promising photosensitizer formulation for PDT.

Few studies comprehensively assessed the progression of melanoma murine models. Here, we have established an advanced melanoma murine model validated by several clinical criterions in terms of tumor size (≈50 mm^3^), Breslow thickness (≈3 mm) and pigmented lesions. In addition, IHC staining for melanoma markers HMB45 and S‐100B further identifies that malignant tumor cells spread through lymphoid organs from established primary tumor. Next, we evaluate the in vivo anti‐tumor activity of CAH. In contrast to palliative effects of chemotherapy and immunotherapy, we find CAH‐mediated PDT completely eradicates the primary tumors and also purges metastatic tumors. Consequently, 90% of cures are achieved and confirmed by histopathological tissue analyses. This curative outcome is also monitored by serum LDH and S‐100B concentrations predicting a low risk of recurrence. Consistently, relapse‐free survival rate of CAH (+) exceeds 80% in 300 days. These results suggest CAH‐mediated PDT may exert a robust systemic immunostimulatory effect to eliminate remaining or recurring melanoma cells, since PDT is active only within the limited illumination area and CAH‐derived PpIX is restricted to tumor‐surrounding area. Additionally, T cells mediated adaptive immune responses play a vital role in the induction of long‐term immune memory to combat recurrence. To test our hypothesis, we first analyze intratumoral T populations showing an elevated level of tumor‐infiltrating T cells in mice receiving CAH (+) compared to that of control (+) and PD‐1 treated mice. Notably, CD4^+^CD8^+^ double positive T subsets are highly activated upon CAH (+); however, no significant changes are noted in the frequency of either CD4^+^ or CD8^+^ T cells among experimental groups. Likewise, splenic T cells display a similar differentiation pattern with enhanced CD4^+^CD8^+^ double positive T populations, although the proportion of T cells in total splenocytes is not altered. Interestingly, a remarkable reduction of T cells expressing neither CD4 nor CD8 surface molecules is observed, suggesting CD4^+^CD8^+^ double positive T cells are more likely to derive from this phenotype. Although this finding appears to contradict with previous studies that CD4^+^CD8^+^ double positive T cells originated from either CD4^+^ or CD8^+^ lineages,[[qv: 20b]] high heterogeneity existing in CD4^+^CD8^+^ double positive T cells may explain their multiple origins.[[qv: 20a]] So far, the function and role of CD4^+^CD8^+^ double positive T cells in the adaptive immune responses still remain to be determined. Normally, mature T cells co‐expressing CD4 and CD8 are rarely detected in peripheral sites, but can be highly augmented to contribute to the inflammatory process under several pathological conditions.^21^ This study demonstrates ≈90% of CD4^+^CD8^+^ double positive T cells display memory phenotypes which may contribute to resistance to tumor recurrence. CD4^+^CD8^+^ double positive T cells are confirmed to produce elevated levels of TNF‐α in response to melanoma compared with CD8^+^ T cells suggesting a cytotoxic potential,^30^ while another study also shows CD4^+^CD8^+^ double positive T cells possess an enhanced capacity to secret proinflammatory cytokines including TNF‐α and IFN‐γ. In line with these published works, we also find serum TNF‐α and IFN‐γ levels are significantly increased upon CAH (+) treatment, although we do not have direct evidence that CD4^+^CD8^+^ double positive T cells are involved in this elevation. These T subsets may have dual functionality in exerting cytolytic as well as helper activities, and become high producers of cytokines with immunomodulatory potential. To our knowledge, this is the first report that PDT induces the activation and proliferation of CD4^+^CD8^+^ double positive T cells, although many studies show more CD8^+^ T cells frequently present in malignant sites after PDT treatments.^31^ We speculate this activation signals may partly correlate with PDT‐induced skin inflammation.^32^ Combining with previous investigations, our preliminary data indicate CD4^+^CD8^+^ double positive T cells may represent a greater cytotoxic T cell phenotype in boosting systemic anti‐tumor immunity compared with CD8^+^ or CD4^+^ single positive T cells; however, their origin and contributions to immune response are still needed to fully elucidate. Intriguingly, we observe the recovery of immune balance in cured mice after withdrawal of CAH, as evidenced by similar T cells differentiation and cytokines levels compared with healthy mice. Thus, CAH‐mediated PDT not only reconstitutes the immune function to battle with disseminated malignant cells, but also maintains a favorable safety profile.

## 4 Conclusions

In summary, our work presents a topically applied PDT nanoformulation (CAH) to cure advanced melanoma in murine models. CAH is capable of overcoming multiple biological barriers for selective delivery of 5‐ALA to melanoma cells. Concurrently with a robust destruction to primary melanoma, CAH‐mediated PDT treatment elicits durable and systemic immunological effects to combat metastases and recurring tumors depending on the activation of CD4^+^CD8^+^ double positive T cells. As a result, a striking curative rate of 90% and relapse‐free survival rate of 83% are achieved without immune‐related adverse effects. Thus, such a cost‐effective treatment without adjuvant immunotherapy might have the great potential to be used clinically for advanced melanoma.

## 5 Experimental Section

*Chemicals and Antibodies: N*,*N*ʹ‐carbonyldiimidazole was obtained from Aldrich. α‐Cyclodextrin was purchased from Shandong Zhiyuan Biotechnology (China). PAMAM‐G2 was synthesized as previously described.[[qv: 12b]] HA (M.W. 200–400 kDa, Cosmetic grade) was obtained from Bloomage Freda BioPharm (China). 5‐ALA hydrochloride was purchased from MedChem Express (USA). Pharmaceutical‐ and cosmetic grade cream bases composed of lecithin, Vaseline and glycerol were purchased from Baixiaotang (China). (4,5‐Dimethylthiazol‐2‐yl)‐2,5‐diphenyl tetrazolium bromide (MTT), dimethyl sulfoxide (DMSO), DMEM medium, fetal bovine serum (FBS), and trypsin were obtained from Gibco (Canada). Cy5 mono NHS ester was obtained from GE Healthcare (UK). LysoTracker Green DND‐26 and MitoTracker Green FM were obtained from Life Technologies (USA). Annexin V‐FITC apoptosis detection kit was purchased from BestBio Biology (China). Fluorometric intracellular ROS kit was purchased from Sigma‐Aldrich (USA). Anti‐mouse PD‐1(CD279) was purchased from Bioxell Life Sciences (USA). APC‐R700 anti‐mouse CD45 antibody, FITC anti‐mouse CD3e antibody, PE anti‐mouse CD8a antibody, APC‐H7 anti‐mouse CD4 antibody, APC anti‐mouse CD44 antibody, and PE‐Cy7 anti‐mouse CD62L antibody were purchased from BD Pharmingen (USA). Mouse anti‐melanoma antibody [HMB45+M2‐7C10+M2‐9E3] was purchased from Abcam (UK). Rabbit anti‐Ki‐67 and rabbit anti‐S‐100B were purchased from Bioss Biology (China). Mouse soluble protein‐100 (S‐100) ELISA kit, mouse interleukin 2 (IL‐2) ELISA kit, mouse interleukin 6 (IL‐6) ELISA kit, mouse tumor necrosis factor (TNF‐α) ELISA kit, and mouse interferon gamma (IFN‐γ) ELISA kit were purchased from Cusabio (USA).

*Preparation and Characterization of CAH*: CDG2 was synthesized according to previously described method by the laboratory.[[qv: 12b]] CAH was formed by following procedure. Briefly, freeze‐dried CDG2 (126.23 mg) was suspended into 5 mL dH_2_O, while 5‐ALA (160 mg) was dissolved in a 10 mL of dH_2_O and added dropwise into CDG2 solution. Then the mixture solution was vigorously stirred and protected from light for 24 h at room temperature, followed by centrifugation at 12 000 rpm for 20 min. The supernatant was collected to measure nonencapsulated 5‐ALA using ultraviolet/visiblespectrophotometer (new Jenway Model 6315) at 670 nm. And the precipitate of ALA/CDG2 complexes resuspended into 5 mL of dH_2_O, and further added with varying amounts of HA (15 mL, dH_2_O) under constant stirring with protection from light for 12 h at room temperature. The resultant solution was centrifuged at 12 000 rpm for 20 min to precipitate CAH which was finally obtained using freeze‐drying under vacuum. The particle size and zeta potential of CAH in dH_2_O were determined with a Zetasizer NS 90 (Malvern Instruments, UK). Transmission electron microscopy (Tecnai Spirit, FEI, USA) was used to observe the morphology of CAH.

To prepare fluorescent Cy5‐labeled CAH, Cy5 NHS ester (0.62 mg, 0.001 mmol) was dissolved in anhydrous DMSO at a concentration of 10 mg mL^−1^, and then was added dropwise to 2 mL CAH solution (8.0 mg, 0.1 m NaHCO_3_, pH 8.3–8.5). The molar ratio of primary amino groups of CAH to NHS esters was 12. After reaction at 4 °C for 24 h in the dark, the mixture was dialyzed against deionized water in a dialysis bag (MWCO, 3.5 kDa) for 3 days, and consequently the product was collected after freeze‐drying.

*Cell Culture*: Murine melanoma cells B16, human melanoma cells A375 and human immortalized the epidermal cells HaCaT were purchased from Laboratory Animal Center of Sun Yat‐sen University (obtained from American Type Culture Collection (ATCC)) and cultured in DMEM medium containing 10% fetal bovine serum in a humidified incubator at 37 °C with 5% CO_2_.

*Measurements of Cellular PpIX Generation*: Murine melanoma cells B16, human melanoma cells A375 were seeded at a density of 8 × 10^3^ cells per well in 96‐well plates for 24 h culture. Then, the medium was replaced with 100 µL of fresh medium containing 5‐ALA or CAH complexes at varying molar ratios of amine to carboxyl groups. Each well maintained a constant 5‐ALA concentration of 8 µg mL^−1^. Cells were treated with blank medium which served as a control. After different time points during a 24 h incubation period, intracellular 5‐ALA was converted to PpIX. In order to determine the intracellular PpIX production, culture medium was removed, and the cells were washed three times with 100 µL PBS. Subsequently, DMSO was added to the extracted cellular PpIX. The fluorescent intensity of PpIX was measured using a spectrofluorometer (EXL800, Bio‐Tek, USA) with an excitation wavelength of 405 nm and an emission wavelength of 635 nm.

To identity the accumulation sites of 5‐ALA or CAH‐derived PpIX in melanoma cells, cells were pretreated with 5‐ALA or CAH containing equal amounts of 5‐ALA (8 µg mL^−1^). After 12 h incubation, the treated cells were further stained with LysoTracker Green or MitoTracker green (final concentration, 100 ×10^−9^ m) according to manufacturer's protocol. Afterward, extracellular fluorescent signal was quenched with 200 µL of Trypan blue (4 mg mL^−1^) followed by washing with three times with cold 1 × PBS. Finally, cells were kept in Phenol red‐free DMEM medium and immediately observed using confocal laser scanning microscopy (FV3000, Olympus, Japan). The images were further analyzed with Image J (NIH, USA).

*Intracellular Localization of CAH*: Murine melanoma cells B16 or human melanoma cells A375 were seeded in 15 mm laser confocal dish at a density of 1.5 × 10^5^ cells per dish, and allowed to culture for 24 h. Subsequently, culture medium was replaced with fresh medium containing Cy5‐labeled CAH with a 5‐ALA concentration of 8 µg mL^−1^. After 4 h incubation, the medium was discarded and the cells were washed thrice with 1 × PBS. And cells were stained with LysoTracker Green (final concentration, 100 × 10^−9^ m) for 30 min at 37 °C. After wash steps, the cells were immediately observed using a confocal microscope (FV3000, Olympus, Japan). The images were further analyzed with Image J (NIH, USA).

*In Vitro Release Assay of CAH*: 150 µg CAH was dissolved in 1.1 mL of aqueous solution at various pHs (7.4, 6.5, 5.5). Three replicates of each sample were evaluated at each time point. At a certain timepoint, each sample was centrifuged at 12 000 rpm for 20 min. The released amounts of 5‐ALA in the supernatant were detected using fluorescamine‐based fluorescence assay.^33^ Briefly, 100 µL of the supernatant at each time point was mixed with 100 µL of 0.1% fluorescamine acetonitrile solution and 100 µL of borate buffer (pH = 8), and allowed the reaction for 10 min at room temperature. Then 100 µL of mixture was immediately measured using a microplate reader under the condition that the excitation wave was 408 nm and the emission wave was 480 nm.

*Cellular Uptake Assay*: Murine melanoma B16 cells and human melanoma A375 cells were seeded in 12‐well plates at a density of 1.5 × 105 cells per well for 24 h culture. Next, the cells were pretreated with or without endocytic inhibitors filipin (10 µg mL^−1^) or CPZ (20 × 10^−6^ m) for 20 min. Then the cells were added with Cy5‐labeled CAH containing 8 µg mL^−1^ of 5‐ALA for 4 h incubation, and were harvested for FACS measurements (Beckman, USA). All experiments detected at least 10 000 cells, and the data were analyzed with software FlowJo (FlowJo LLC, USA).

*Cytotoxicity Test*: B16, A375 or HaCaT cells were cultured at a seeding density of 8 × 10^3^ cells per well in a 96‐well plate for 24 h. To test phototoxicity of laser power, the cells were irradiated with 635 nm laser (MRL‐III‐635L Red Diode Laser, Changchun New Industries Optoelectronics Tech, China) at various light density for 10 min. Likewise, cytotoxicity was measured after the cells were cultured with CAH or free 5‐ALA for 12 h under various conditions. The cells were continually cultured for another 48 h before MTT assay.

*Detection of ROS In Vitro*: ROS generation in vitro was determined using a Reactive Oxygen Species Assay Kit following the supplier's procedure. Briefly, cells were plated at a seeding density of 1.5 × 10^5^ cells in a laser confocal dish for 24 h culture, and subsequently exposed to 8 µg mL^−1^ of 5‐ALA or CAH for 12 h. Cells were washed thrice with 1 × PBS and then stained with 2ʹ,7ʹ‐dichlorofluorescein diacetate (DCFH‐DA, 10 × 10^−6^ m) for 20 min. After various illumination experiments, ROS production was imaged with 495 nm excitation and 535 nm emission using a confocal microscope (FV3000, Olympus, Japan). ROS quantification was performed using ImageJ software (NIH, USA) according to a formula to calculate the corrected total cell fluorescence (CTCF). CTCF = Integrated Density − (Area of selected cell × Mean fluorescence of background readings).

*Apoptotic/Necrotic Assay*: Annexin V‐FITC/PI apoptosis detection kit was used to evaluate the cell apoptotic/necrotic rate following the manufacturer's instructions. After exposed to 5‐ALA or CAH for 12 h, B16 and A375 cells were irradiated with 635 nm at a light dose of 25 mW cm^−2^ for 5 min and 85 mW cm^−2^ for 3 min, respectively. After wash steps with 1 × PBS, cells were stained with 10 µL of annexin V‐FITC for 15 min followed by staining with 20 µL of PI for 5 min in the dark. Discarding the staining solution, cells were observed under digital inverted microscope (EVOS, Fisher Scientific, USA). To quantify apoptotic/necrotic rate, the annexin V‐FITC/PI double‐stained cells were harvested and washed for FACS analysis (FACS‐Calibur Instrument, Beckman, USA). The data was analyzed with Summit software (Version 5.2, Beckman, USA).

*Ex Vivo Skin Permeation Study*: A system equipped with Franz diffusion cells was employed for skin permeation study. Abdominal hair of male Sprague Dawley (SD) rats with weighs of 200–250 g was shaved using electric and hand razors. After anesthetizing the rats with ether, the abdominal skin was surgically excised and removed the fat tissue carefully. The excised rat skin was set in place with the stratum corneum facing the donor compartment and the dermis facing the receptor, each cell had a diffusion area of 3.14 cm^2^. The receiver compartment was filled with 7.5 mL of PBS (pH 5.0) containing 0.1% formaldehyde as a preservative, and was stirred magnetically in a circulating water bath with a constant temperature of 37 °C to keep the skin surface at ≈33 °C. free 5‐ALA or CAH containing the same amount of 5‐ALA were incorporated in a conventional cream formulation (20% w/w, 25 mg cream) were topically applied to skin. After 12 h, the dissolution samples were collected from the receiver compartment to determine the permeated 5‐ALA concentration using fluorescamine‐based fluorescence assay.

*TEWL Measurement*: The barrier integrity of C57BL/6 mice skin was examined using TEWL measurements. The dorsal hair on tumor sites was shaved 24 h prior to applying drug‐containing cream. After drug administration for 12 h, all formulations were removed. Subsequently, TEWL was quantitatively measured using a Tewameter 210 (Courage & Khazaka, Germany). The TEWL values were calculated automatically and expressed as g cm^−2^·h in terms of base units.

*Establishment of An Advanced Melanoma Murine Model*: Male/female C57BL6J mice with 4–5 weeks of age were purchased from the Laboratory Animal Center of Sun Yat‐sen University (Guangzhou, China). All in vivo studies were carried out in accordance with the Guide for Care and Use of Laboratory Animals which were approved by the Institution Animal Care and Use Committee of Sun Yat‐sen University. To establish an advanced melanoma murine model, the mice were subcutaneously injected on the dorsal flank with 1 × 10^6^ B16 cells, which were presuspended in 200 µL RPMI 1640 medium containing 30% of Matrigel. Maximum (L) and minimum (W) was measured with a slide caliper every two days to monitor the tumor growth. Tumor volume can be calculated as (width^2^) × (length) × 0.5.

*Antitumor Test*: After tumor inoculation of 3 days, tumor volume reached 40–100 mm^3^. Then the mice bearing B16 tumors were randomly divided into five groups (*n* = 10), which were further treated with 20% w/w 5‐ALA cream, 20% w/w CAH cream, blank cream, PD‐1, DTIC, respectively. For light‐based PDT, various cream formulations were topically applied on tumor sites with hair‐shaved every two days constituting seven dosages. After 12 h drug administration, the mice were anesthetized with 1% pentobarbital sodium, and the tumors were subsequently irradiated with a 635 nm laser at a light dose of 25 mW cm^−2^ for 5 min. Anti‐PD‐1 antibody was i.p. injected at a dose of 200 µg per mouse every three day constituting three dosages, while DTIC were i.v. injected at a dose of 10 mg kg^−1^ every day constituting ten dosages. Tumor size and body weight were measured with a digital caliper and balance every two day, respectively. During the treatment period, blood was collected from each mouse every four days. Blood samples were immediately centrifuged at 3000 rpm for 20 min to harvest serum, which was used to detect LDH, S‐100B, IL‐2, IL‐6, TNF‐α, and IFN‐γ concentrations with the corresponding assay kits. Serum biochemistry assay was performed by an automatic biochemistry analyzer ((Hitachi 3100, Hitachi High Technologies, Inc., Japan), while complete blood count was determined with Sysmex XT‐2000iV (IDEXX BioResearch, Japan).

After 15 days posttreatments, CAH (+)‐cured mice were kept alive, while the rest of uncured mice were sacrificed for histological analysis. Subsequently, tumors and all the organs and tissues including brain, heart, liver, spleen, pancreas, lung, kidney and inguinal and mesenteric lymph nodes were excised, weighed, washed with saline, photographed, and finally fixed in the 4% paraformaldehyde solution. After 2 days of fixation, all tissues were embedded in paraffin blocks. The sections (6 µm) were cut from each block, and stained with H&E and immunohistochemical markers (Ki67, HMB45, S‐100B) for microscope observation (EVOS FL Auto, Life Technologies, USA).

To calculate the survival rate, all the treated mice described above and healthy mice (*n* = 10) were weighed and monitored regularly until death occurred or euthanasia was performed.

*Biodistribution of CAH‐Derived PpIX In Vivo*: After being topically applied with 20% w/w CAH, blood samples were collected at 4 and 12 h. Then the mice were sacrificed, tumors and surrounding skins were excised for further PpIX detection. Tumors were homogenized in 2 mL of ethyl acetate/glacilacetic acid (4:1) solution for 30 min extraction, and then centrifugated at 3000 × *g* for 30 min. The supernatant was collected for further detections. Skins were sheared into tiny pieces and added with 2 mL of extraction solvent which consisted of DMSO and methanol (1:1, v/v). Subsequently, PpIX was extracted from skin by using an ultrasonic apparatus which was operated at 40% amplitude for 30 min in the dark. Then the mixture solution was centrifuged for 30 min at 3000 × *g* and supernatant was collected. All samples were measured using a fluorescent spectrophotometer with excitation and emission wavelengths of 405 and 635 nm, respectively.

*Fluorescent Detection of ROS and PpIX in Tumor and Skin*: To investigate the ROS generation in vivo, B16 tumor‐bearing mice were randomly divided into 3 groups (*n* = 3). when the tumor volume reached 100 mm^3^, 20% w/w 5‐ALA cream, 20% w/w CAH cream and blank cream were applied topically. After 12 h posttreatment, B16 tumor‐bearing mice were anesthetized and the tumors were exposed to a 635 nm laser with a photodensity of 25 mW cm^−2^ for 5 min. For PpIX detection in vivo, the mice were exposed to blank cream, 20% CAH‐containing cream for 12 h without laser irradiation. Then the mice were sacrificed and tumors with surrounding skin were collected and frozen, which were further cut into sections of 6 µm thickness. The sections were further stained with DAPI and DCFH‐DA and imaged with confocal microscope.

*FACS Measurements for Immune Response*: After 4, 8, and 14 days of drug administration, tumor, spleens, inguinal lymph nodes, were harvested. Single‐cell suspensions were created by mechanical dissociation with 70 µm cell strainers (BD Biosciences, USA). Cells were immediately stained with the following fluorochrome‐conjugated antibodies anti‐CD45, anti‐CD3e, anti‐CD4, anti‐CD8a, anti‐CD44 and anti‐CD62L according to manufacturer's protocol, which were further analyzed with a flow cytometer (FACS Cantotm II, BD Biosciences, USA). Data analysis was carried out using FlowJo software.

*Statistical Analysis*: Statistical analysis was performed using GraphPad Prism (GraphPad Software, Inc., USA). Statistical comparisons were performed using one‐way analysis of variance (ANOVA) followed by Bonferroni's post hoc test for multiple comparisons. Data were obtained from at least three independent samples, and expressed as mean ± standard deviation (S.D.) unless otherwise noted and *p* < 0.05 was considered significant.

## Conflict of Interest

The authors declare no conflict of interest.

## Supporting information

Supplementary
