# Supplementary material for: Robust Photodynamic Therapy Using 5‐ALA‐Incorporated Nanocomplexes Cures Metastatic Melanoma through Priming of CD4+CD8+ Double Positive T Cells
Source: Adv Sci (Weinh). 2019 Jan 20;6(5):1802057. doi: 10.1002/advs.201802057 (PMC6402398; doi:10.1002/advs.201802057)
Supplement: Supplementary file 1 — Supplementary [file ADVS-6-1802057-s001.pdf]

Copyright WILEY-VCH Verlag GmbH & Co. KGaA, 69469 Weinheim, Germany, 2019.

ADVANCED SCIENCE Open Access

Supporting Information

for *Adv. Sci.,* DOI: 10.1002/advs.201802057

Robust Photodynamic Therapy Using 5-ALA-Incorporated Nanocomplexes Cures Metastatic Melanoma through Priming of CD4^{+}CD8^{+} Double Positive T Cells

Zhi Li, Cuifeng Wang, Huihui Deng, Jiamin Wu, Huan Huang, Ran Sun, Hongbo Zhang, Xiaoxing Xiong, and Min Feng**

Supporting Information

Robust Photodynamic Therapy Using 5-ALA-incorporated Nanocomplexes Cures Metastatic Melanoma through Priming of CD4^{+}CD8^{+} Double Positive T cells

Zhi Li[#], Cuifeng Wang[*#], Huihui Deng, Jiamin Wu, Huan Huang, Ran Sun, Hongbo Zhang, Xiaoxing Xiong, Min Feng[*]

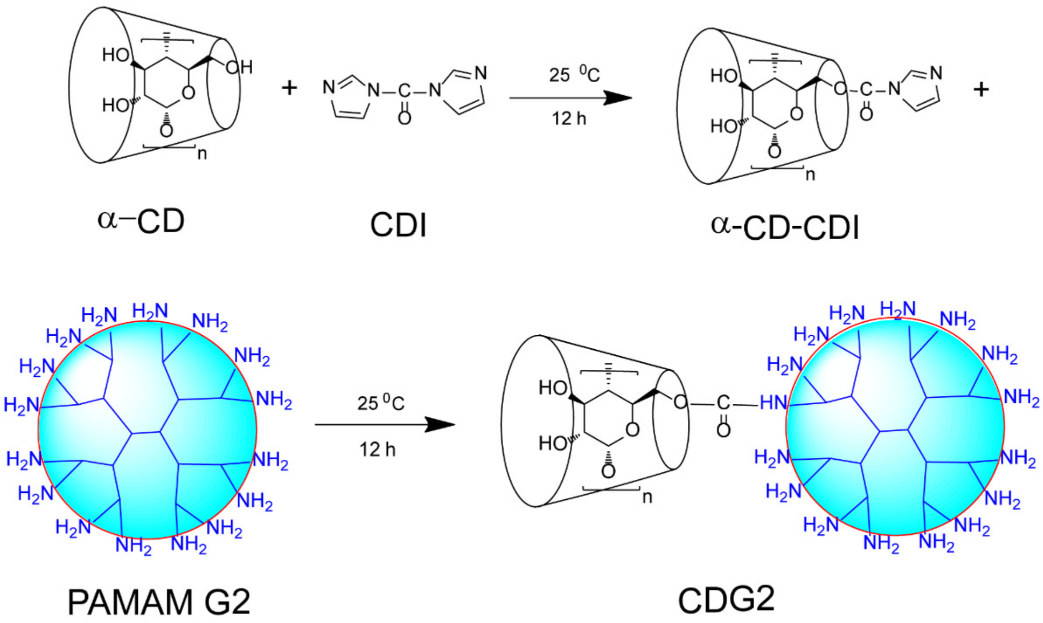

Figure S1 Synthetic scheme of CDG2.

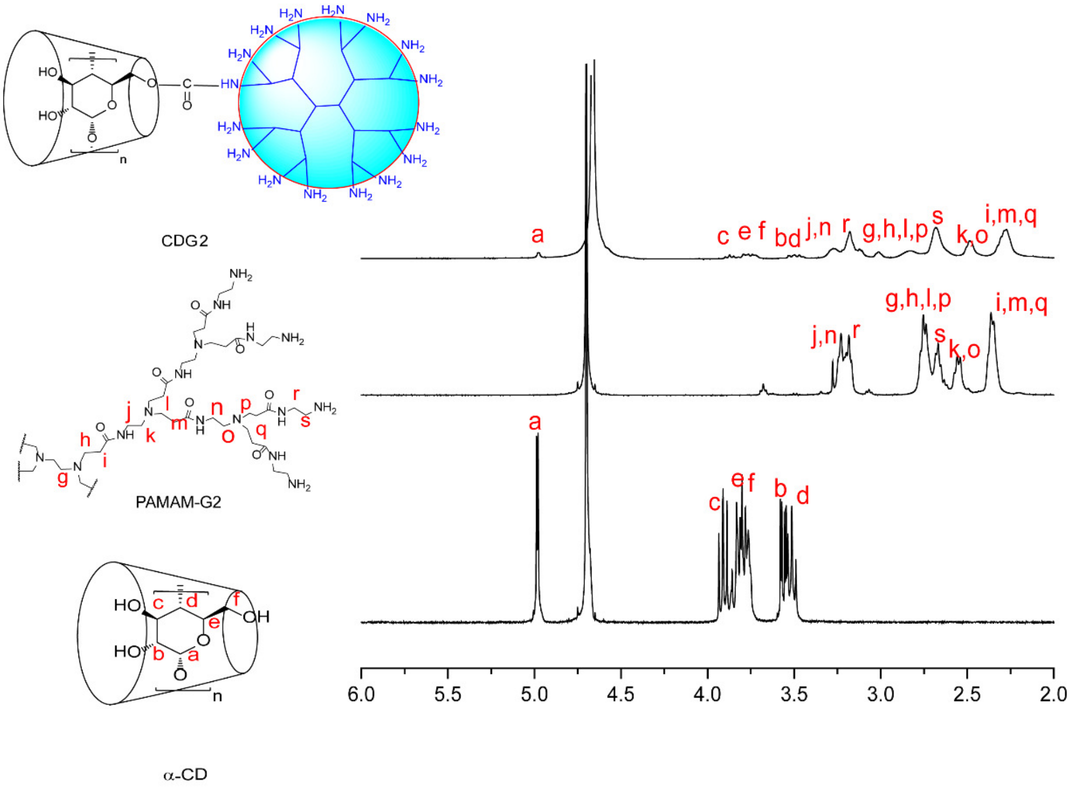

Figure S2 NMR spectrum of CDG2, PAMAM-G2 and α-CD.

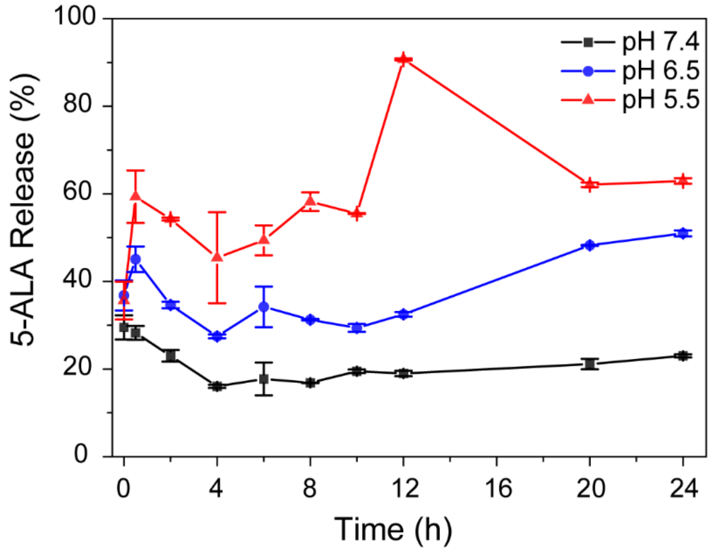

Figure S3 In vitro release profiles of 5-ALA from CAH at various pH buffers over a period of 24 h. All data were expressed as mean ± S.D. of three independent samples.

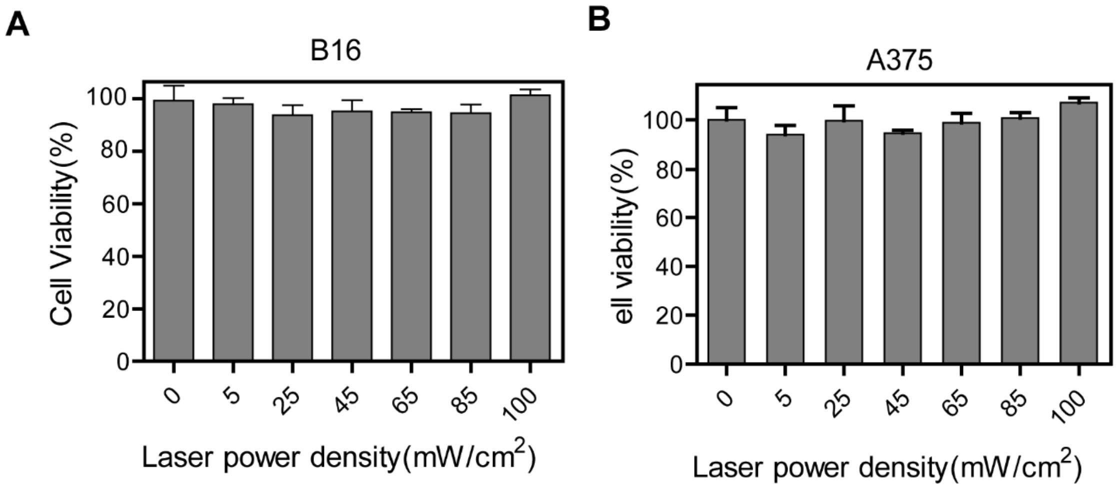

Figure S4 Photocytotoxicity was exerted by a range of light does in B16 cells **(A)** and A375 cells **(B)**. All data were reported as mean ± S.D. of three independent experiments.

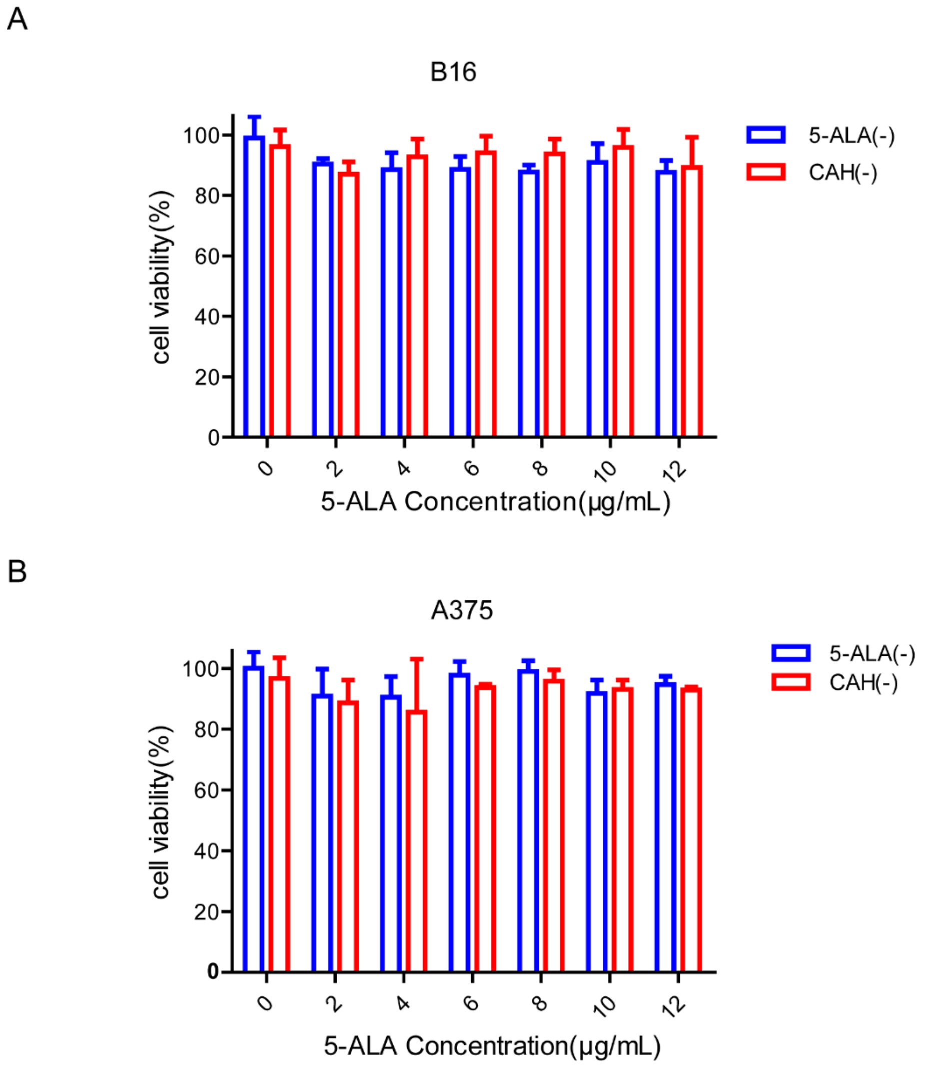

Figure S5 Cytotoxicity of CAH without light irradiation (-) was evaluated in B16 cells **(A)** and A375 cells **(B)** by MTT assay. The same amount of 5-ALA was used as control. All data were reported as mean ± S.D. of three independent experiments.

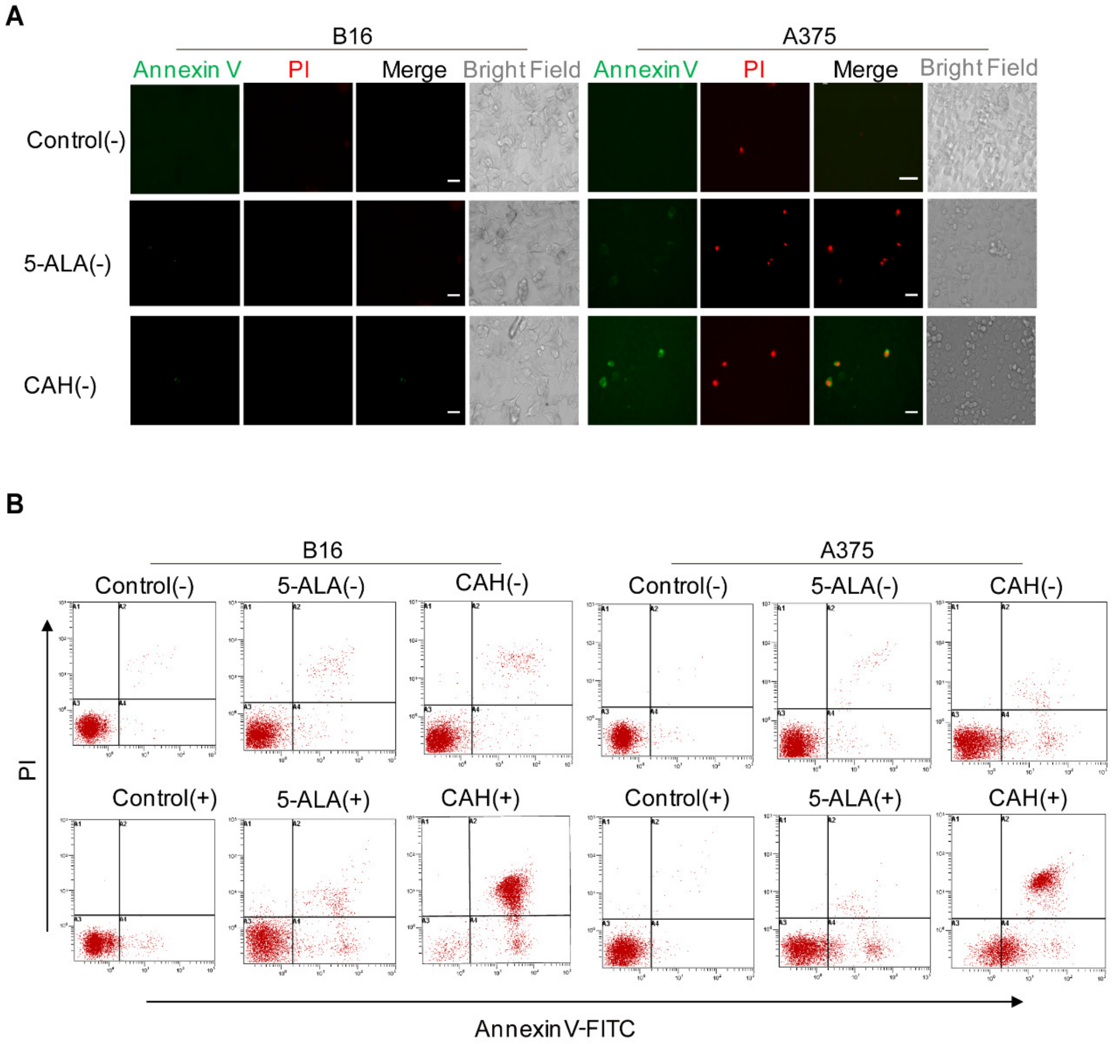

Figure S6 (A) B16 cells or A375 cells were treated with 5-ALA or CAH for 12 hrs without light illumination (-). Then apoptotic cells were detected by Annexin V-FITC/PI double-staining. Scale bar was 50 μm. **(B)** Cell apoptotic rate was measured using flow cytometry followed by various treatments. Annexin V-FITC$^+$ PI$^-$ (lower right quadrant) or Annexin V-FITC$^+$ PI$^+$ (upper right quadrant) staining demonstrated B16 cells and A375 cells undergoing the early or late period of apoptosis. All results were representative of three independent experiments.

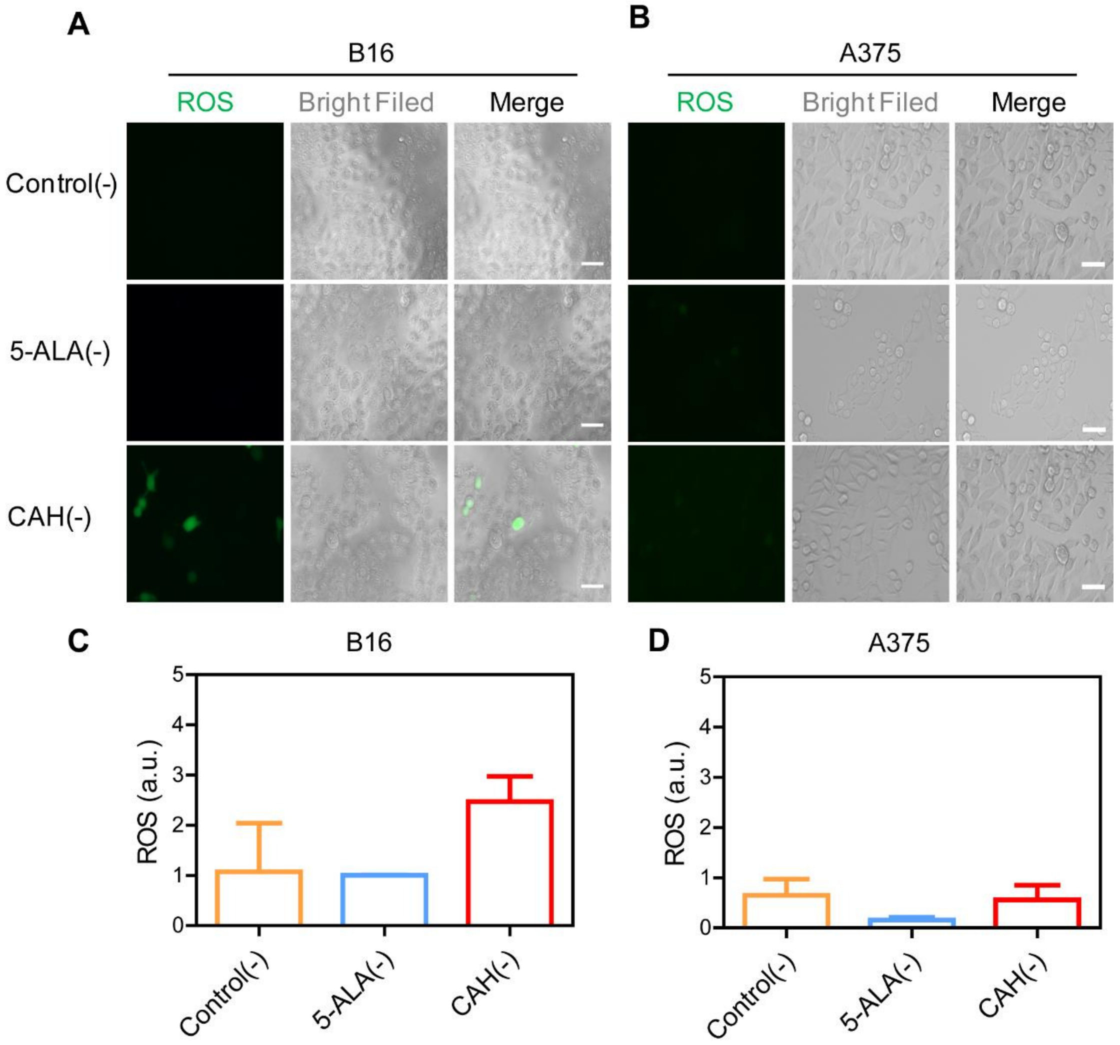

Figure S7 (A-B) Intracellular ROS (green) production of 5-ALA or CAH in the absence of light (-) was imaged with a fluorescent microscope, **(C-D)** and fluorescent intensity was further quantified using a fluorometer. The data were shown as the mean values for four samples ± S.D.

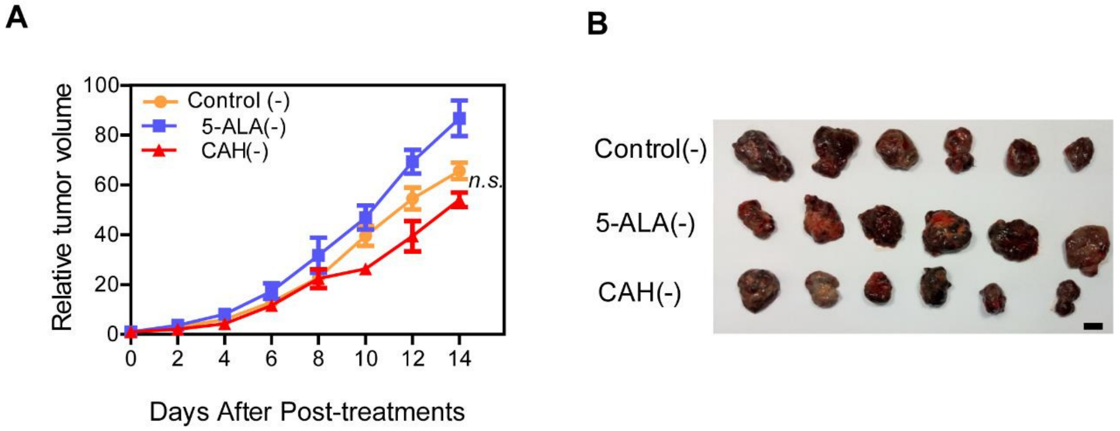

Figure S8 Relative tumor volume of the mice receiving various treatments without light illumination was monitored over time. All the data were shown as mean ± S.D., n =10, n.s., not significant vs control (-).

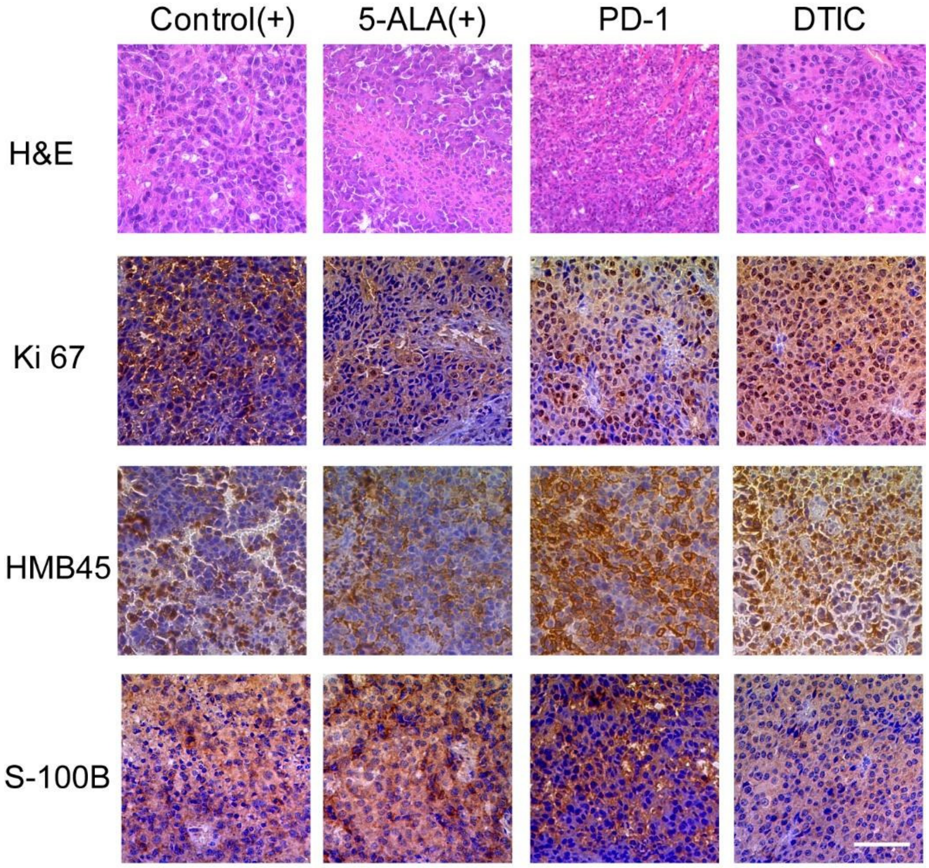

Figure S9 H&E staining and IHC staining with antibodies to Ki67, HMB45 and

S-100B. Scale bar was 50 μm.

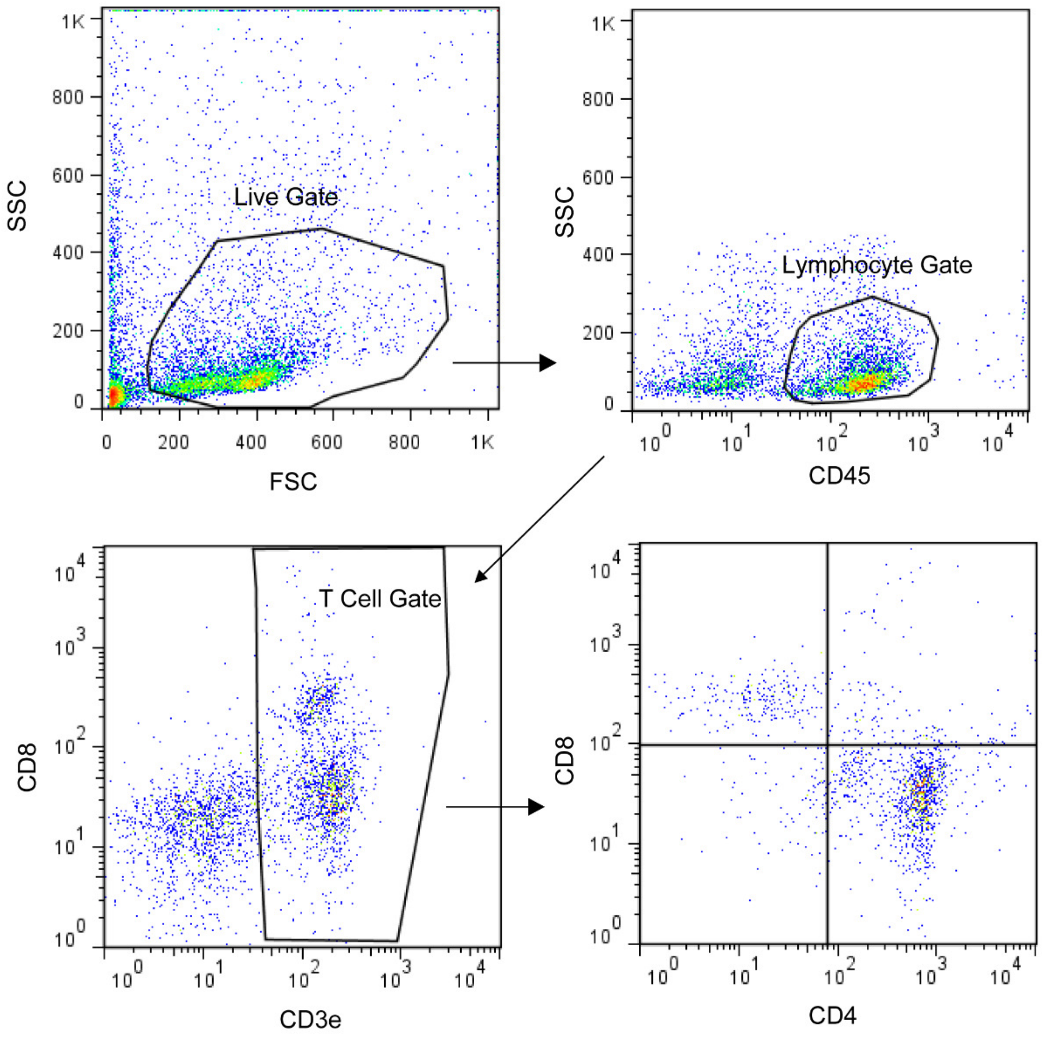

Figure S10 An example of the gating strategy was used to distinguish $CD4^+$, $CD8^+$, and $CD4^+CD8^+$ T cells within tumor, sentinel lymph node, and spleen from each tested mouse.

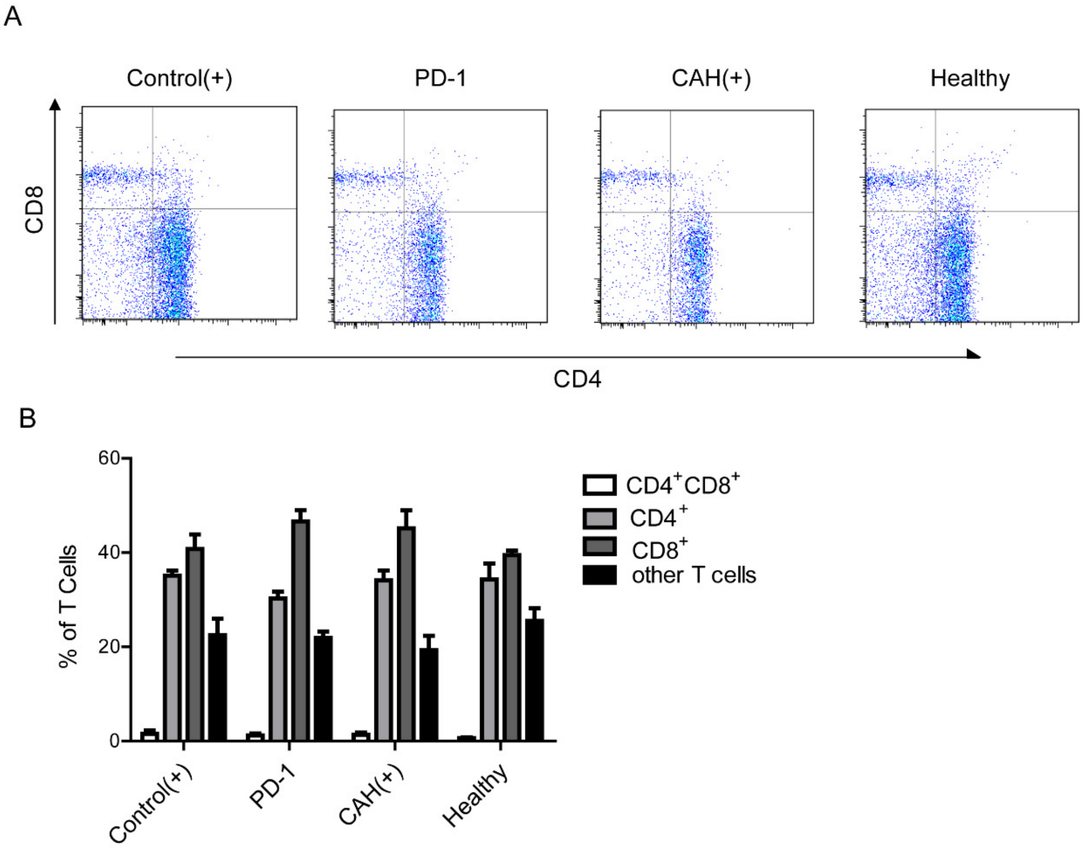

Figure S11 Frequency and phenotype of circulating T cells in the healthy mice and the mice treated with control (+), PD-1 and CAH (+). All the data were shown as mean ± S.D., n = 3-5 mice per group.

Table S1 Elemental analysis of CDG2.

Element	C (%)	H (%)	N (%)
	48.37	8.75	21.20
